# Supplementary material for: Feasibility and acceptability pilot of video-based direct observed treatment (vDOT) for supporting antitubercular treatment in South India: a cohort study
Source: BMJ Open. 2023 May 29;13(5):e065878. doi: 10.1136/bmjopen-2022-065878 (PMC10230861; doi:10.1136/bmjopen-2022-065878)
Supplement: Supplementary data [file bmjopen-2022-065878supp001.pdf]

Table 1: Mixed-effects multilevel logistic regression analysis of the determinants of daily responsiveness during follow–up

**Variable**		**Null Model**	**Logistic regression**	**Final Model**
Fixed Effect Model		**Adjusted Odds Ratio (95% Confidence Interval)**	**Adjusted Odds Ratio (95% Confidence Interval)**	**Adjusted Odds Ratio (95% Confidence Interval)**
Sex	Females		0.99 (0.65-1.5)	0.55 (0.04 -8.36)
	Males		*Reference*	*Reference*
Age			1.04 (1.03 – 1.06)	1.04 (0.95 -1.14)
Area of residence	Urban		0.76 (0.09-6.43)	
	Rural		*Reference*	
Marital status	Married		5.09 (1.32 -19.65)	
	Unmarried		*Reference*	
Employment status	Employed		0.13 (0.06 -0.25)	0.01 (0.0003 – 0.31)
	Unemployed		*Reference*	*Reference*
Family income	Above Poverty Line		0.82 (0.54 – 1.26)	
	Below Poverty Line		*Reference*	
Literacy	Literate		0.18 (0.06 - 0.51)	0.04 (0.0007 -2.70)
	Illiterate		*Reference*	*Reference*
Owns a phone	Yes		2.16 (0.7 – 6.7)	
	No		*Reference*	
Site of TB	Pulmonary TB		0.42 (0.28 -0.63)	0.37 (0.03 -4.63)
	Extra Pulmonary TB		*Reference*	*Reference*
Patient Preference of vDOT	Prefers vDOT over DOT		40.08 (11.75 – 136.67)	
	Prefers DOT over vDOT		*Reference*	
Time from treatment initiation to enrolment			0.97 (0.97 – 1.06)	0.97 (0.95 – 1.14)
Random Effect Model				
At individual level				2.19 (1.48 - 3.26)
Intra class cluster coefficient (95%CI) at Individual level		0.70 (0.51 - 0.84)		0.59 (0.40 – 0.76)
Akaike Information Criterion (AIC)		1129	1446	1130
