# Supplementary material for: Feasibility and acceptability pilot of video-based direct observed treatment (vDOT) for supporting antitubercular treatment in South India: a cohort study
Source: BMJ Open. 2023 May 29;13(5):e065878. doi: 10.1136/bmjopen-2022-065878 (PMC10230861; doi:10.1136/bmjopen-2022-065878)
Supplement: Author's manuscript [file bmjopen-2022-065878.draft_revisions.pdf]

BMJ Open

BMJ Open is committed to open peer review. As part of this commitment we make the peer review history of every article we publish publicly available.

When an article is published we post the peer reviewers' comments and the authors' responses online. We also post the versions of the paper that were used during peer review. These are the versions that the peer review comments apply to.

The versions of the paper that follow are the versions that were submitted during the peer review process. They are not the versions of record or the final published versions. They should not be cited or distributed as the published version of this manuscript.

BMJ Open is an open access journal and the full, final, typeset and author-corrected version of record of the manuscript is available on our site with no access controls, subscription charges or pay-per-view fees (http://bmjopen.bmj.com).

If you have any questions on BMJ Open's open peer review process please email info.bmjopen@bmj.com

BMJ Open

Feasibility and acceptability of a video – based treatment adherence monitoring system in South India – A cohort pilot study

Journal:	*BMJ Open*
Manuscript ID	bmjopen-2022-065878
Article Type:	Original research
Date Submitted by the Author:	04-Jul-2022

Complete List of Authors:	Rodrigues, Rashmi; St John's National Academy of Health Sciences, Community Health; Karolinska Institute, Public Health<br>Varghese, Suman; St John's National Academy of Health Sciences, St. John's Research Institute<br>Mahrous, Mohammed; Independent Researcher<br>Ananthaneni, Anil Kumar; St John's National Academy of Health Sciences, St. John's Research Institute<br>Ahmed, Mohammed; St John's National Academy of Health Sciences, St. John's Research Institute; Blackpool Teaching Hospital, NHS trust<br>D'Souza, George; St John's National Academy of Health Sciences, Pulmonary Medicine

Keywords:	Public health < INFECTIOUS DISEASES, INTERNAL MEDICINE, Epidemiology < INFECTIOUS DISEASES

SCHOLARONE™
Manuscripts

I, the Submitting Author has the right to grant and does grant on behalf of all authors of the Work (as defined in the below author licence), an exclusive licence and/or a non-exclusive licence for contributions from authors who are: i) UK Crown employees; ii) where BMJ has agreed a CC-BY licence shall apply, and/or iii) in accordance with the terms applicable for US Federal Government officers or employees acting as part of their official duties; on a worldwide, perpetual, irrevocable, royalty-free basis to BMJ Publishing Group Ltd ("BMJ") its licensees and where the relevant Journal is co-owned by BMJ to the co-owners of the Journal, to publish the Work in this journal and any other BMJ products and to exploit all rights, as set out in our licence.

The Submitting Author accepts and understands that any supply made under these terms is made by BMJ to the Submitting Author unless you are acting as an employee on behalf of your employer or a postgraduate student of an affiliated institution which is paying any applicable article publishing charge ("APC") for Open Access articles. Where the Submitting Author wishes to make the Work available on an Open Access basis (and intends to pay the relevant APC), the terms of reuse of such Open Access shall be governed by a Creative Commons licence – details of these licences and which Creative Commons licence will apply to this Work are set out in our licence referred to above.

Other than as permitted in any relevant BMJ Author's Self Archiving Policies, I confirm this Work has not been accepted for publication elsewhere, is not being considered for publication elsewhere and does not duplicate material already published. I confirm all authors consent to publication of this Work and authorise the granting of this licence.

For peer review only

Title: Feasibility and acceptability of a video – based treatment adherence monitoring system in South India – A cohort pilot study

Authors: Rashmi Rodrigues[1,2,3](rashmijr@gmail.com), Suman Sarah Varghese[4] (sumansarah.92@gmail.com), Mohammed Mahrous[5] (mohammed.mahrousdr@gmail.com), Anil A. Kumar[4] (anil_kumarans@yahoo.co.in), Mohammed Naseer Ahmed[4,6] (dr.m.naseer.ahmed@gmail.com), George A. D'Souza[7] (dsouza.ga1975@gmail.com)

1. Department of Community Health, St. John's Medical College, Bangalore, Karnataka, India
2. Department of Global Public Health, Karolinska Institutet, Stockholm, 10435, Sweden
3. DBT/Wellcome Trust India Alliance, Hyderabad/New Delhi, India
4. St. John's Research Institute, Bangalore, Karnataka, India
5. Independent researcher
6. Blackpool Teaching Hospital, NHS trust
7. Department of Pulmonary Medicine, St. John's Medical College, Bangalore, Karnataka, India

Corresponding Author:

Dr. Rashmi Rodrigues
Associate Professor, Department of Community Health,
St. John's Medical College, Bangalore -560034
Email id: rashmijr@gmail.com
Phone number: 9845389538

Word Count: 4372

For peer review only

ABSTRACT

Objectives: The objective of this study was to determine the feasibility and acceptability of a video–based anti-Tuberculosis treatment adherence monitoring system in South India.

Design: A cohort pilot study

Study setting: Participants were recruited at the Tuberculosis (TB) treatment center of a tertiary level teaching facility in Bangalore, Karnataka, South India.

Participants: The study enrolled 25 patients with TB (PwTB), with replacement. Adult PwTB who were on drug sensitive treatment regimens. Those who had drug resistant TB were excluded from the study.

Intervention: Participants received scheduled adherence reminders and were trained to video-record themselves swallowing their medication via a video DOT (vDOT) mobile application. The application was automated to submit these videos for evaluation. Participants were followed-up monthly till treatment completion.

Outcome Measures: Adherence rate and acceptability of vDOT.

Results: The mean age of the participants was 33 (±14) years, majority were females (16, 64%), resided in urban areas (24,96%), married (17, 68%) and had access to smart phones (23,92%). A total of 3247 person-days of follow – up was completed; of the videos submitted (2675), 93% (2488/2675) were considered 'acceptable' and sixteen (64%) participants were optimally adherent (i.e.,≥80%). Participant videos improved in quality and a higher proportion met acceptability criteria over time. Twenty-one (84%) participants stated that they found the application easy to learn; 13 (52%) preferred vDOT over DOT. Exact logistic regression did not show any statistically significant associations between demographic features and optimal adherence.

Conclusion: Video-based mobile phone interventions are acceptable to patients with TB and the ease of using the application increases with time. To provide patient centered care, vDOT is a promising option that can be offered to patients for treatment support and adherence monitoring.

Key words: mHealth, Pilot study, Treatment monitoring, Tuberculosis, video-based DOT

Article Summary:

- The vDOT application allowed asynchronous uploading of videos which improved its utility in situations where there are interrupted internet services.
- Issues with the application and challenges faced by participants were identified and addressed.
- Though the application had the potential for reporting of adverse events (AEs), this was not enabled to minimize the complexity of the intervention. Participants, however, contacted the research team via mobile phones when necessary.
- The vDOT application reduces the amount of patient – provider contact. However, this drawback was overcome by regular SMS communications and calls incase of a missed dose.

For peer review only

Introduction:

Globally, ten million people are diagnosed with tuberculosis annually with India accounting for approximately a quarter of these cases (1). This, despite the availability of Anti-Tuberculosis Treatment (ATT), comprising antimicrobials and evidence based preventive strategies (2). Further, Tuberculosis (TB) is distinctive as it impacts the working population and has sizeable economic ramifications (2).

In India, TB has an incidence of TB is 164 per 100000 population, with 87% newly diagnosed, 10% previously treated, and 2.8% Multi–Drug Resistant (MDR) patients with TB in 2020, while, 82% were successfully treated (3). ATT generally lasts six months and nonadherence to treatment is the leading cause for the emergence drug resistance– impacting costs of care, treatment duration and outcome (4).

In order to address the challenges with adherence to ATT, Direct Observed Treatment Short–course (DOTS) was adopted globally as a feasible solution (5). Direct Observed Treatment (DOT), the fulcrum of the DOTS strategy, provides the much-needed follow–up as well as definitive evidence that medications were taken in the prescribed dose and manner. DOT, however, is not devoid of challenges. Facility based DOT requires patients to visit the treatment provider and often results in loss of time and wages. On the other hand, community-based DOT runs the risk of compromising confidentiality due to health workers frequently visiting patients, leading to stigma. Further, such a strategy is human resource intensive from a programme perspective. These concerns, and the current ubiquity of digital communication via mobile phones have spurred innovative alternatives to DOT (5).

India has a tele-density of 87%, which can be harnessed to address challenges in TB management (6). Telehealth is a useful as a tool in educating, identifying new cases, and monitoring adherence (10). The significant digitalization of India in the past decade, with the availability of quality internet services at affordable prices, presents itself as a viable resource to meet healthcare needs online or wirelessly (7). mHealth advances designed to monitor ATT range from simple Short Message Service (SMS) reminders to sophisticated technology with ingestible sensors (8). The 'Nikshay Sampark' call–center under the National TB Elimination Programme (NTEP) uses Interactive Voice Response (IVR) and SMS services as adherence reminders (8). The 'real time Medication Event Reminder Monitor (rtMERM),' an electronic pill box with pressure sensors, detects the weight of the pills in the box and provide alarms as reminders to patients (9–12). The NTEP also uses the 'Patient Compliance toolkit' a mobile based application along with a smart card, through which phone interventions such as medication reminders, TB health tips, follow up calls, and financial incentives are delivered (8).

Furthermore, the "99DOTS", an adherence monitoring strategy was rolled out during the Covid 19 pandemic when DOT was logistically challenging. For this, patients give a missed call to a toll-free phone number that is revealed when the pills are popped from the ATT blister pack (13).

The renewed commitment of the government through the NTEP in the backdrop of the Sustainable Developmental Goals makes the development of a solution for poor treatment adherence expedient. 99DOTS offers the advantage of avoiding unnecessary travel, privacy and remote monitoring (14). In comparison, a video-based monitoring tool offers definitive evidence that the patient has in fact consumed the medication in the prescribed manner. The value of these, digital innovations for adherence support, were realized during the COVID 19 pandemic, when DOT was impossible and remote monitoring the only viable option. Given the ubiquity of mobile phones, remote monitoring of ATT via mobile phones is a suitable alternative to DOT (12). In this backdrop, our study provides valuable evidence on the feasibility and acceptability of a video–based treatment adherence monitoring system (vDOT) among patients with pulmonary tuberculosis on Anti-Tuberculosis Treatment (ATT) in South India.

For peer review only

Methods:

We carried out a cohort pilot study to assess the role of video DOT in adherence monitoring at a tertiary care, referral teaching hospital in Bangalore, India. Participants recruited for the study were aged >18 years newly diagnosed with drug sensitive tuberculosis, had at least 2 months of treatment pending, and were willing upload videos of themselves swallowing their ATT via the video DOT (vDOT) mobile application daily. Patients with HIV co-infection, recurrent TB or drug resistant TB were excluded from the study. Recruitment was done with replacement.

Study setting

St. John's Medical College Hospital (SJMCH) is a tertiary care, referral and teaching hospital that provides emergency, outpatient, inpatient, community outreach, and day care services through its 1350 bedded hospital. It is equipped with laboratory, imaging and interventional facilities including radiography, smear microscopy, mycobacterial culture, and nucleic acid amplification test (NAAT, GeneXpert). Patient diagnosed with TB are referred for treatment to the DOTS centre at the hospital that functions through a public private partnership with the NTEP. PwTB receive treatment from the NTEP through the DOTS centre gratis. Alternatively, PwTB at SJMCH can also avail treatment for a cost via the hospital's pharmacy.

Standard Care

Treatment is given in accordance with national guidelines using standard regimen for drug sensitive TB. It prescribes a daily treatment regimen based on the patient's weight. It consists of 2 months of Intensive Phase (IP) in which the patient takes 4 drugs [Isoniazide (H), Rifampicin (R), Pyrazinamide (Z), Ethambutol (E)] followed by 4 months of Continuous Phase (CP) where 3 drugs (H,R,E) are taken in Fixed Dose Combination (FDC) pills.

Conventionally, the patient is expected to visit a health care provider 6 days a week and swallow the medications under their direct supervision. These visits are documented on a DOTS card. In case of a missed dose, the treatment is lengthened till the required number of doses are completed. Adherence monitoring at the time of the study, was via in-person DOTS. Currently, all PwTB on ATT at the DOTS center receive 99DOTS.

The intervention: Video DOT (vDOT)

For vDOT, participants receive an adherence reminder via a vDOT mobile application (v.Cure, described below), installed on their mobile phone. Participants use the application to video–record themselves while taking their medication. The application is automated to upload the recorded videos when connected to the internet.

The uploaded videos are reviewed by trained research assistants at the back end of the application, who classify the videos as *accepted* (dose taken) or *rejected (video received but dose not taken)*. The videos are accepted if the participant can be identified, the pills are shown before swallowing, and the pill is swallowed under adequate lighting. If not, the video is deemed to be *"rejected"*. Participant is considered to have *"missed"* videos if no video is received for the day. Participants who miss videos are contacted via phone on the subsequent.

"Acceptability" of the vDOT application was assessed using an interviewer administered questionnaire at the end of the study. The assessed design of the application –functionality (i.e. speed, ease of learning to use the application, clarity of options, consistency of interactions across all components), aesthetics (i.e. arrangement and size of buttons/icons, its resolution and overall visual appeal), the participants' opinion (if they would

For peer review only

recommend the application to others and if they would pay for the application) and rating of the application was based on a five– star rating system. Participants experiences with the application – regarding reminders, privacy, ease of using the application were also assessed.

The vDOT mobile Application

User interface: A smartphone friendly application developed for vDOT was v.Cure, available at the Google Play Store (https://play.google.com/store/apps/details?id=in.stjhons) in five languages, English, Kannada, Hindi, Tamil and Telugu, each in their own script. The application is restricted to users in India. The participant logs into the application, installed on their mobile phones, using their credentials, and is able to view a instructional video that guides them through the application, and demonstrates how to capture and upload video. Participants may skip the instructional video and access the recording screen directly. After recording the video, the participant is informed of the status of the video, i.e., pending/uploaded. The participant chooses the language for the application as well as the time for the adherence reminder notification while registering the application.

The web interface consists of participant demographic and clinical profiles, well as their submitted video linked to their adherence calendar. The research staff view the video and indicate if the video is accepted or rejected. The adherence calendar reflects the status of the videos (accepted, rejected, missed), and also communicates the status to the participant via the v.Cure application. (Fig 1)

Participant Enrolment and Follow-up

At baseline, trained research assistants administered consent, obtained relevant demographic and clinical information and installed the v.Cure mobile application the participants mobile phone. If the participant did not own a mobile phone but wished to participate in the study, a phone was provided. The participant was then trained to use the mobile application and upload videos of themselves taking their medication.

Follow-ups were monthly and combined with pill refill visits, pill counts and clinical assessments. The v.Cure database helped monitor adherence. The videos received were labelled accepted, rejected or missed, by trained research assistants. Reasons for missed videos were obtained via phone calls or in person visits. Acceptability of the intervention was assessed at the end of the study.

Definitions of outcomes

'Treatment success' - sum of participants cured (smear microscopy negative at the end of treatment) and participants completing treatment (have taken all prescribed doses of ATT without a smear microscopy report).

The 'adherence rate' is the proportion of number of uploaded videos that were accepted to the total number of videos expected (includes both, rejected and missed). 'Optimal adherence' - an adherence rate $\geq$ 80%.

Intervention Fidelity

All the participants who fulfilled the selection criteria and consented to participate in the study were trained in the use of vDOT application – registration, logging in, recording and uploading videos. Participant concerns at this time were addressed. Further, phone numbers of study personnel were provided to participants to call in the event of a challenge with the application. The research team viewed the videos that were uploaded daily. Participants who missed videos or submitted erroneous videos were contacted and their concerns recorded and addressed.

For peer review only

Analysis

Treatment adherence recorded via the v.Cure application was made available in Microsoft Excel format. The data obtained at baseline and follow-up's form participants collected entered in a Microsoft Excel database created for the purpose and analysed using STATA v12.0. Data was described using frequencies and measures of central tendency and deviation as applicable. Participant experiences with the vDOT intervention were similarly described.

Treatment adherence (optimal adherence ≥80%) was the outcome assessed. Subsequent to a bivariate analysis, variables associated with the outcome at p=0.20 were included in an *exact*-binary logistic regression to identify associations between the socio-demography, clinical factors and optimal adherence. Adjusted and unadjusted Odds Ratios are reported.

Patient and Public involvement

This was a part of a larger trial that uses vDOT as an adherence monitoring and treatment support tool (CTRI no: CTRI/2017/07/009052). Prior to the development of the intervention, acceptability of such an intervention among PwTB was conducted in Bangalore city and in Ujjain. (15,16) Patients' suggestions and concerns were taken into consideration during the design of the trial and during the development of the v.Cure application.

Results:

A total of 258 patients were screened, of whom 150 were eligible, and 25 were initially enrolled in the study. These 25 participants contributed 3247 person days of follow-up to the study.

Not owning/not interested in learning to use a smartphone [37(34%)] and not interested in research [20(18%)] were the most common reasons for declining to participate in the study (Table 1).

Of the initial 25 participants enrolled, 15 participants dropped out within two months of enrollment and were replaced; the most common reason for withdrawal was concern regarding privacy and stigma (5, 33%).

Table 1: Reasons for replacing participants after enrollment and for refusing consent

Sl. No.	Reasons cited	Number (%)
Unwilling to participate		**N=110**
1	Not willing to learn to use a smartphone/not interested in research	57(52%)
2	Stigma and Privacy Issues	22(20%)
3	Time constraints	14(13%)
4	Other Health Issues	6(5%)
5	Family not comfortable	4(4%)
6	Do not believe they have TB	3(3%)
7	Would need to depend on others	2(2%)
8	Network issues at home	2(2%)
Participants who were replaced		**N=15**
1	Withdrew consent after participants' families were concerned of stigma and privacy	5 (33%)
2	Participants were unable to upload videos	3 (20%)
3	Participants were dependent on someone else to either take videos or upload them	3 (20%)
4	Lost to follow – up after two months of initiation of the intervention	3 (20%)
5	Network issues	1 (7%)

For peer review only

Demographic characteristics of the 25 study participants who completed 6 months is given in the Table 2. Sixteen (64%) participants were female and 9 (36%) were male. Most participants had an urban background (96%), were married (68%), and had access to a smartphone (92%). The median income among the participants was □15,000 (IQR 7,000 – 22,750).

Table 2: Socio–demographic and clinical features

Factor	Level	Female	Male	Total	p-value
N		16	9	25	
Age, mean (SD)		33 (15)	34 (12)	33 (14)	0.887
Family income*, n (%)	BPL	6 (38%)	7 (78%)	13 (52%)	0.097
	APL	10 (62%)	2 (22%)	12 (48%)	
Residence, n (%)	Rural	0 (0%)	1 (11%)	1 (4%)	0.280
	Urban	16 (100%)	8 (89%)	24 (96%)	
Marital status, n (%)	Single	6 (38%)	2 (22%)	8 (32%)	0.661
	Married	10 (62%)	7 (78%)	17 (68%)	
Children, n (%)	Yes	9 (56%)	6 (67%)	15 (60%)	0.691
	No	7 (44%)	3 (33%)	10 (40%)	
Access to a Smartphone, n (%)	No	2 (13%)	0 (0%)	2 (8%)	0.520
	Yes	14 (87%)	9 (100%)	23 (92%)	
Owns a phone, n (%)	No	1 (6%)	0 (0%)	1 (4%)	1.000
	Yes	15 (94%)	9 (100%)	24 (96%)	
TB site, n (%)	Pulmonary	4 (25%)	6 (67%)	10 (40%)	0.087
	Extra Pulmonary	12 (75%)	3 (23%)	15 (60%)	
Completed study#, n (%)	No	3 (19%)	0 (0%)	3 (12%)	0.66
	Yes	13 (81%)	9 (100%)	22 (88%)	

#Dropouts:3, 2nd month 2, 3rd month 1; *according to WHO standards

Adherence

Total number of videos received was 82% (2675 videos received of 3247 expected videos based on prescribing dosing frequency). Among submitted videos, 93% (2488/2675) met the criteria to be considered 'acceptable' (Fig. 2).

All 25 participants had at least one missed recorded dose. While 604 missed entries were recorded, participants could not be contacted in nearly two-fifths (238) of the instances. Among those contacted (366, 61%), 'technical issues' with the application was cited as the most common reason for not uploading the video (Table 3).

Table 3: Reasons for not uploading (missed) videos among those who were contacted

**Sl. No.**	**Reasons**	**n (%) N=366**
1	Technical issues with the application and phone	69 (19%)
2	Claimed to have uploaded	63 (17%)
3	Travel – personal or health related	45(12%)
4	Lost phone/ phone repair	31 (8%)
5	Shared Phone/dependent	26 (7%)
6	Forgot to upload Video	25 (7%)
7	No Network	21 (6%)
8	Wants to withdraw	19 (5%)

For peer review only

9	No power	13 (4%)
10	Forgot to take medicines	12 (3%)
11	Change in schedule/phase	11 (3%)
13	Still Learning the App	9 (3%)
12	Accidentally deleted the app	8 (2%)
14	In Person DOTS	7 (2%)
15	No Permission from DOTS provider	7 (2%)

Sixteen (64%) participants had an optimal adherence rate through vDOT of ≥ 80% (mean adherence 91%, ± 6%). The overall average adherence rate was 70% ± 26%, ranging from 12-99%. The remainder had less than 80% of videos accepted (12 participants, mean adherence 51% ± 18%).

Exact logistic regression

Participants who were female, those married, those literate, those gainfully employed, those above poverty line socio-economic status, and those who preferred DOT were more adherent to medication, none of these associations were found to be statistically significant (Table 4). This shows that demographic factors do not play a significant role in achieving optimal adherence rates using the video DOT application. (Table 4)

Table 4: Comparison between adherence ratio and demographic variables

Variable		Adherence rates		Unadjusted Odds ratio (95% Confidence Interval)	Adjusted Odds ratio (95% Confidence Interval)
		Optimal	Sub-Optimal		
Sex	Female	06	03	1.20	1.41
	Male	10	06	(0.16 – 12.3)	(0.13 – 18.31)
Age [mean ±SD]		33.94 ±15.94	31.78 ±10.2	1.01 (0.95 – 1.08)	1.03 (0.99 – 1.11)
Employment Status					
	Unemployed	07	04	0.63	0.76
	Employed	09	05	(0.09 – 4.27)	(0.06 – 9.61)
Socio-economic status	BPL	09	05	1.03	0.82
	APL	07	04	(0.14 – 7.04)	(0.08 – 6.33)
Literacy					
	Illiterate	02	01	0.88	1.25
	Literate	14	08	(0.01 –19.52)	(0.01 – 178.63)
Marital Status					
	Unmarried	06	02	0.49	0.28
	Married	10	07	(0.04 – 3.94)	(0.004 – 6.22)
Participant preference	vDOT	08	05	1.24	0.47
	DOT	08	04	(0.18 – 8.87)	(0.01 – 12.21)

Acceptability and feasibility

While none of the participants reported that the application was broken, majority of them reported perfect functioning. Eleven (44%) participants faced some challenges with accuracy of features and components, and speed of the application features and components. Most participants stated that they were either able to use the application immediately [11(44%)] or it was easy to use [10(40%)]. Majority of the participants found the menu labels/icons/buttons to be perfectly clear [14(56%)]. It was commonly expressed that interactions were perfectly consistent and intuitive across all components and screens (Fig. 3).

For peer review only

All the participants felt that the arrangement and size of the buttons/icons/menus/content on the screen were satisfactory and appropriate. Over half the participants reported that the resolution of the graphics used was of very high quality. Majority stated that the application had very high visual appeal (Fig. 3).

Privacy and application related preferences

Most of the participants were not worried about discrimination due to TB (84%). While 2 (8%) participants found vDOT intrusive (8%), most participants found the passcode feature effective in protecting their privacy [20(80%)]. A quarter of the participants stated that the mobile application had been accessed by others in the preceding month and nearly all participants reported that someone had seen them recording the video. However, none of these instances were followed by a negative experience. Most participants preferred recording the videos themselves. Less than two – fifths of the participants found the application difficult to maneuver once opened. (Fig. 4)

Intervention Fidelity

The most common reason cited for not uploading a video was "technical issues with the application" at both, the front and back end (69, 19%). These problems (table 5) were resolved after discussion with application developers. An interesting issue identified was incompatibility of the application with a few lower-end smart phone models. This was resolved by adjusting settings in the Google playstore, while mobile phone models that continued to be incompatible with the application were not used in the study. Participants with such phones or those without phones were given a smart phone for the purpose of participation.

Table 5: Issues identified with the vDOT Application

Sl. No.	Issue raised
Participant perspective	
1	The pop – up reminders intended to prompt participants to take medications were not working
2	In case participants forgot their passcode to access the application, they would contact the team and a new pin would be generated.
Research team perspective	
3	The application was not compatible with two particular smartphone models
4	The application was designed to automatically calculate the 'acceptance ratio', however it was not being done accurately.
5	Once the video that was uploaded by the participant had been reviewed, the participant list would get reshuffled
6	The back-end screen would get stuck after rejecting a video
7	In spite of the participants uploading the videos, it could be viewed only after 1 –2 days
8	The missed entries were not sorted by any particular order, we requested the application developers to sort them by date
9	Missed doses were not captured in the data tracker calendar for individual participants
10	Percentage adherence, at times, exceeded 100%

Discussion

This study sought to assess whether using a video–based application to deliver DOTS was acceptable and feasible for treatment adherence support for ATT. Nearly two – thirds (16, 64%) of the participants had an optimal adherence rate of over 80% with no other socio – demographic factors associated with optimal adherence. Most participants reported that they found the application easy to use with a high visual appeal.

For peer review only

We found that the most common reasons for not participating were unwillingness to learn how to use a smartphone and disease related stigma. The known barriers to mHealth are poor internet connectivity, security and privacy concerns and English literacy (17) and privacy concerns were also noted in our study. Given that the application was video based it required participants to learn the skill of opening the application and recording the video- which does not require language literacy. Further, as the population was largely urban internet connectivity was not an issue.

Of the 2675 doses observed on the application, over 90% were of acceptable quality. Nearly two – thirds of the participants had optimal adherence rates. Of the missed doses, 3% were attributed to forgetting to take the dose and 7% forgot to record the video. While, a cross-sectional study done among HIV-TB co-infected patients using 99DOTS, reported that 26% had forgotten to take the medication and 6% participants forgot to make a call after taking the medication (18). This was probably because the preset reminders and calls for missed doses probably helped sustain favorable behavior.

Further, mHealth interventions are expected to improve the quality, accessibility and cost – effectiveness of healthcare interventions available to patients (17). According to the National Strategic Plan for TB (NSP) 2017-2025 a good patient support plan is vital for treatment success and should be developed at the time of treatment initiation (8). Educating patients, families and communities regarding TB and its treatment while improving access to patient centered care which improves treatment adherence and outcomes (17). The vDOT application addresses these needs and hence is a valuable resource.

Being asynchronous, the vDOT has similar benefits as 99DOTS, namely, convenience, decreased absenteeism from work and reduction in travel costs (18). Additionally, logistic difficulties during the manufacturing and supply of customized sleeves for the ATT blister packs used in 99DOT are avoided. On the other hand, ingestible sensor in pills requires the patient to wear wireless wearable readers that capture data from the sensor once the pill is ingested. The wearable readers are usually worn on their wrist, arm, hip or abdomen, which may cause discomfort and which if seen by others can potentially increase stigma. On the other hand vDOT is considered a less intrusive and a more autonomous adherence monitoring tool (19,20) as the participant can use an inconspicuous mobile phone for the purpose. Besides, patients may find ingesting a sensor intrusive (21) and the bulk of the wearable sensor poses as a barrier to its uptake (24).

The application we developed, uses a passcode to authenticate access to the application. When participants forgot their passcode they called the study team to generate a new code. Aside from concerns regarding the authenticity of the caller, this system worked efficiently to provide participants with timely access to the application. However, we did not receive any complaints from the participants stating that others had accessed their phones and generated passcodes.

The limitation of the current version of the application is that it does not support the reporting of adverse events (AEs). The decision not to include this feature was made by the research team to reduce complexity in terms of resources required. Albeit, participants were provided with appropriate contact numbers to reach out to the study team in an emergency. In the future, the application may be modified to include reporting of AEs by patients. The vDOT minimized patient – provider interaction. To overcome this drawback, participants were called whenever a missed dose was recorded, in addition to the monthly visits at the facility.

Interventional fidelity was ensured by uncompromising adherence to the protocol, appropriate training of participants to use the application and timely redressal of challenges identified– which was one of the purposes of the pilot. While patient preferences may depend on familiarity with the mHealth delivery platform (23), our study did not find familiarity with smartphones to be a predictor of adherence. This however may be attributed to the small sample size of the study. The study also shows that patients with little or no exposure to

For peer review only

smart phones can be successfully trained to use the application which was confirmed with the increasing proportion of videos that were accepted increases with time. Overall, while the small sample size limits generalisability, the study has helped identify and address technical challenges with the application and understand its acceptability.

Conclusion

vDOT is an acceptable alternative to conventional DOTS for ATT adherence support and monitoring. The application was easy to learn and nonintrusive, nearly equally proportions of participants preferred vDOT over DOT and vis versa. Though concerns with privacy and stigma limited participation, features like the passcode to access the mobile application helped alleviate participant concerns. However, given the increasing digital literacy and the growing confidence in digital technology in the backdrop of pandemics such as the Covid 19, the use of vDOT for adherence support in tuberculosis is imperative. Yet, with the availability of multiple digital adherence interventions, PwTB may be offered a basket of choices– similar to the "cafeteria approach" for methods of contraception, for treatment adherence support – of which vDOT is one.

Data Availability

The data is made available on open science framework (DOI 10.17605/OSF.IO/Z4S9C)

Data are available under the terms of the Creative Commons Attribution 4.0 International license (CC-BY 4.0).

Funding Statement

This work was supported by DBT/Wellcome Trust India Alliance (number IA/CPHI/15/1/502042) and the Swedish Research Council (number 2015-02750).

Acknowledgements

The authors would like to thank Ms Shubha Krishnamurthy, Ms Hannah DJ, Dr Nikitha Prabhakar, and Dr Naseer Ahmed who supported data collection and data management. The authors also appreciate the support from the offices of the district TB officers, Bruhat Bengaluru Mahanagara Palike, and Bangalore Urban District, Bangalore, India, through their respective public health care facilities in urban and rural Bangalore. We would like to thank Focaloid Technologies for their dedication and approachability that was instrumental in the development of the application.

Conflict of interest

The authors have no conflict of interest.

Author contribution

RR and GD were involved in the concept and design of the study and in monitoring the study, AAK and AN were involved in administering informed consent, data collection, entry and monitoring, MM and SSV were involve in drafting the paper and data analysis, RR was involved in the final revision of the article.

Ethics Statement

Ethics clearance was obtained from the Institutional Ethics Committee, St. John's Medical College, Bangalore (IEC no 342/2016). Written informed consent was obtained from all individuals willing to participate. Participants were provided study related information in a language that they understood. For illiterate participants, all information was imparted orally and thumb impression along with the signature of an impartial witness obtained.

References

1. World Health Organization (WHO). WHO report on TB 2020. Vol. 1, Who. 2020.

2. Rajeshwari R, Balasubramanian R, Muniyandi M, Geetharamani S, Thersa X, Venkatesan P. Socio-economic impact of tuberculosis on patients and family in I...: Ingenta Connect. Int J Tuberc Lung Dis [Internet]. 1999 Oct [cited 2022 Apr 5];3(10):869–77. Available from: https://www.ingentaconnect.com/content/iuatld/ijtld/1999/00000003/00000010/art00005

3. Ministry of Health and Family Welfare. INDIA TB REPORT 2022 - Coming Together to End TB Altogether [Internet]. New Delhi; 2022 [cited 2022 May 11]. Available from: https://tbcindia.gov.in/WriteReadData/IndiaTBReport2022/TBAnnaulReport2022.pdf

4. Central TB Division. India TB Report 2019. Welfare, Ministry of Health and Family. 2019. p. 244.

5. Chauhan LS, Tonsing J. Revised national TB control programme in India. Tuberculosis. 2005 Sep;85(5–6):271–6.

6. Telecom Regulatory Authority of India. No Title.

7. PWC. How mHealth can revolutionise the Indian healthcare industry. PWC, India. 2018;

8. National Strategic Plan for Tuberculosis Elimination 2017-2025 [Internet]. 2017 [cited 2022 Apr 5]. Available from: https://tbcindia.gov.in/WriteReadData/NSP Draft 20.02.2017 1.pdf

9. Liu X, Blaschke T, Thomas B, De Geest S, Jiang S, Gao Y, et al. Usability of a medication event reminder monitor system (MERM) by providers and patients to improve adherence in the management of tuberculosis. Int J Environ Res Public Health. 2017;14(10):1–11.

10. Wang N, Shewade HD, Thekkur P, Huang F, Yuan Y, Wang X, et al. Electronic medication monitor for people with tuberculosis: Implementation experience from thirty counties in China. PLoS One. 2020;15(4):1–14.

11. Hayes TL, Hunt JM, Adami A, Kaye JA. An Electronic Pillbox for Continuous Monitoring of Medication Adherence. Conf Proc IEEE Eng Med Biol Soc [Internet]. 2006 [cited 2022 Apr 5];1:6400. Available from: /pmc/articles/PMC2911441/

12. Thomas BE, Kumar JV, Periyasamy M, Khandewale AS, Mercy JH, Raj EM, et al. Acceptability of the Medication Event Reminder Monitor for Promoting Adherence to Multidrug-Resistant Tuberculosis Therapy in Two Indian Cities: Qualitative Study of Patients and Health Care Providers. J Med Internet Res [Internet]. 2021 Jun 1 [cited 2022 Apr 5];23(6). Available from: https://pubmed.ncbi.nlm.nih.gov/34110300/

13. Government of India. Using Mobile phones to Improve Adherence to to Tuberculosis Medication. Central TB Division of India, Minsitry of Health and Family Welfare. 2021.

14. Oberoi S, Kumar Gupta V, Chaudhary N, Singh A. 99 DOTS. [cited 2022 Jan 10]; Available from: www.ijcmr.com

For peer review only

15. Kumar AA, De Costa A, Das A, Srinivasa GA, D'souza G, Rodrigues R. Mobile Health for Tuberculosis Management in South India: Is Video-Based Directly Observed Treatment an Acceptable Alternative? JMIR mHealth uHealth [Internet]. 2019 [cited 2022 Apr 1];7(4). Available from: /pmc/articles/PMC6468344/

16. Rao JS, Diwan V, Kumar AA, Varghese SS, Sharma U, Purohit M, et al. Acceptability of video observed treatment vs. directly observed treatment for tuberculosis: a comparative analysis between South and Central India. Wellcome Open Res 2022 7152 [Internet]. 2022 May 13 [cited 2022 Jun 18];7:152. Available from: https://wellcomeopenresearch.org/articles/7-152

17. Wipro Council for Industry Research. The mHealth Case in India [Internet]. Bangalore; 2013 [cited 2022 May 19]. Available from: www.wipro.com

18. Ghatage S, M S A, Aithal SS, D B S, Angadi N. A study on reasons for nonadherence to 99DOTS among HIV-tuberculosis coinfected patients in Davanagere district, Karnataka. Int J Med Sci Public Heal [Internet]. 2018;7(10):805–8. Available from: http://www.ijmsph.com/fulltext/67-1528277879.pdf?1641794950

19. Gebreweld FH, Kifle MM, Gebremicheal FE, Simel LL, Gezae MM, Ghebreyesus SS, et al. Factors influencing adherence to tuberculosis treatment in Asmara, Eritrea: A qualitative study. J Heal Popul Nutr. 2018;37(1):1–9.

20. Prabhu A, Agarwal U, Tripathy JP, Singla N, Sagili K, Thekkur P, et al. "99DOTS"techno-supervision for tuberculosis treatment – A boon or a bane? Exploring challenges in its implementation at a tertiary centre in Delhi, India. Indian J Tuberc. 2020 Jan 1;67(1):46–53.

21. Distefano MJ, Schmidt H. mHealth for Tuberculosis Treatment Adherence: A Framework to Guide Ethical Planning, Implementation, and Evaluation. [cited 2022 May 21]; Available from: www.ghspjournal.org

22. Mason M, Cho Y, Rayo J, Gong Y, Harris M, Jiang Y. Technologies for Medication Adherence Monitoring and Technology Assessment Criteria: Narrative Review. JMIR mHealth uHealth [Internet]. 2022 Mar 1 [cited 2022 May 26];10(3). Available from: /pmc/articles/PMC8949687/

23. Kumar K, Loebinger MR, Ghafur S. The role of wirelessly observed therapy in improving treatment adherence. Futur Healthc J. 2022;9(2).

24. Chai PR, Goodman G, Majo B, Mendez L, Yassir M, et al. Design and Delivery of Real-Time Adherence Data to Men Who Have Sex with Men Using Antiretroviral Pre-exposure Prophylaxis via an Ingestible Electronic Sensor. 2020 [cited 2022 May 26]; Available from: https://doi.org/10.1007/s10461-020-03082-y

25. Granger D, Vandelanotte C, Duncan MJ, Alley S, Schoeppe S, Short C, et al. Is preference for mHealth intervention delivery platform associated with delivery platform familiarity? BMC Public Health [Internet]. 2016 Jul 22 [cited 2022 May 26];16(1):1–7. Available from: https://bmcpublichealth.biomedcentral.com/articles/10.1186/s12889-016-3316-2

Figure Legend:

Figure 1: v.Cure Application operation

Figure 2: Proportions of videos that were accepted, missed or rejected over the period of the study

Figure 3: Acceptability of the Application

Figure 4: Concerns regarding privacy and application related preferences

For peer review only

For peer review only

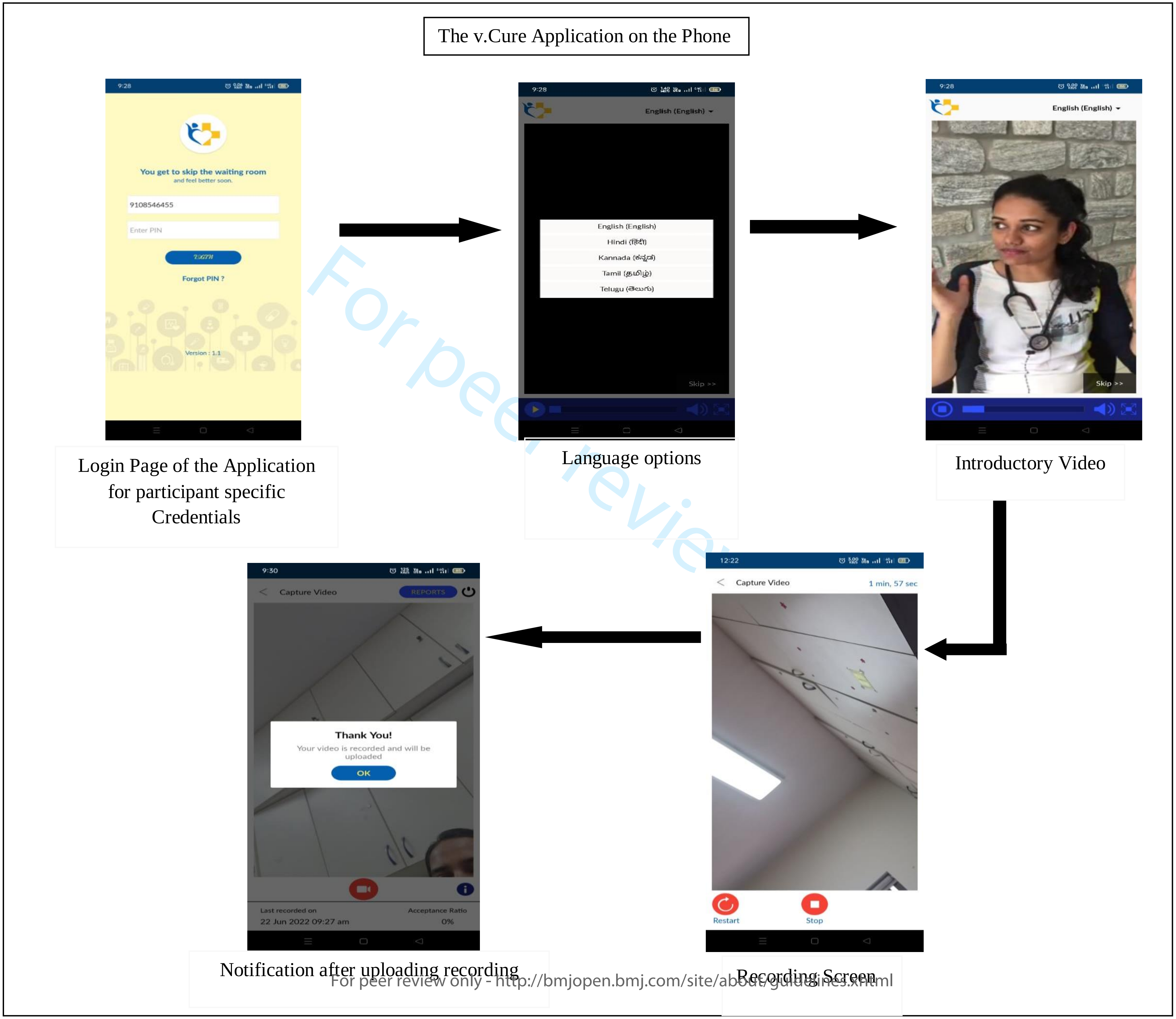

For peer review only - http://bmjopen.bmj.com/site/about/guidelines.xhtml

The v.Cure Application on the Back – end

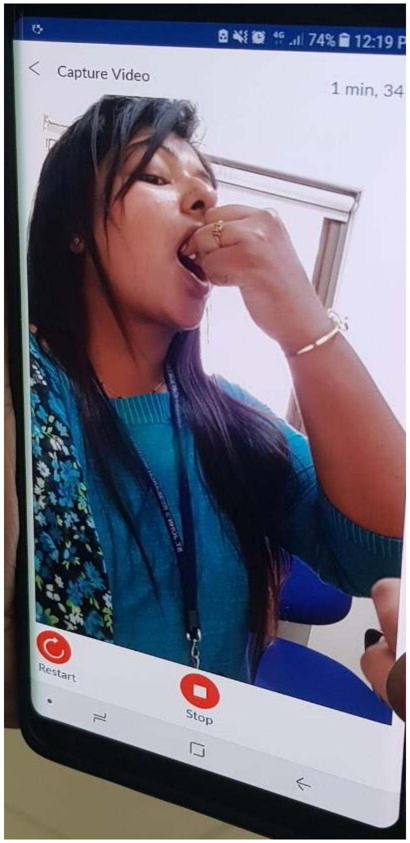

Video recording that is viewed on the back – end of the Application. Actionable: Reject/Accept

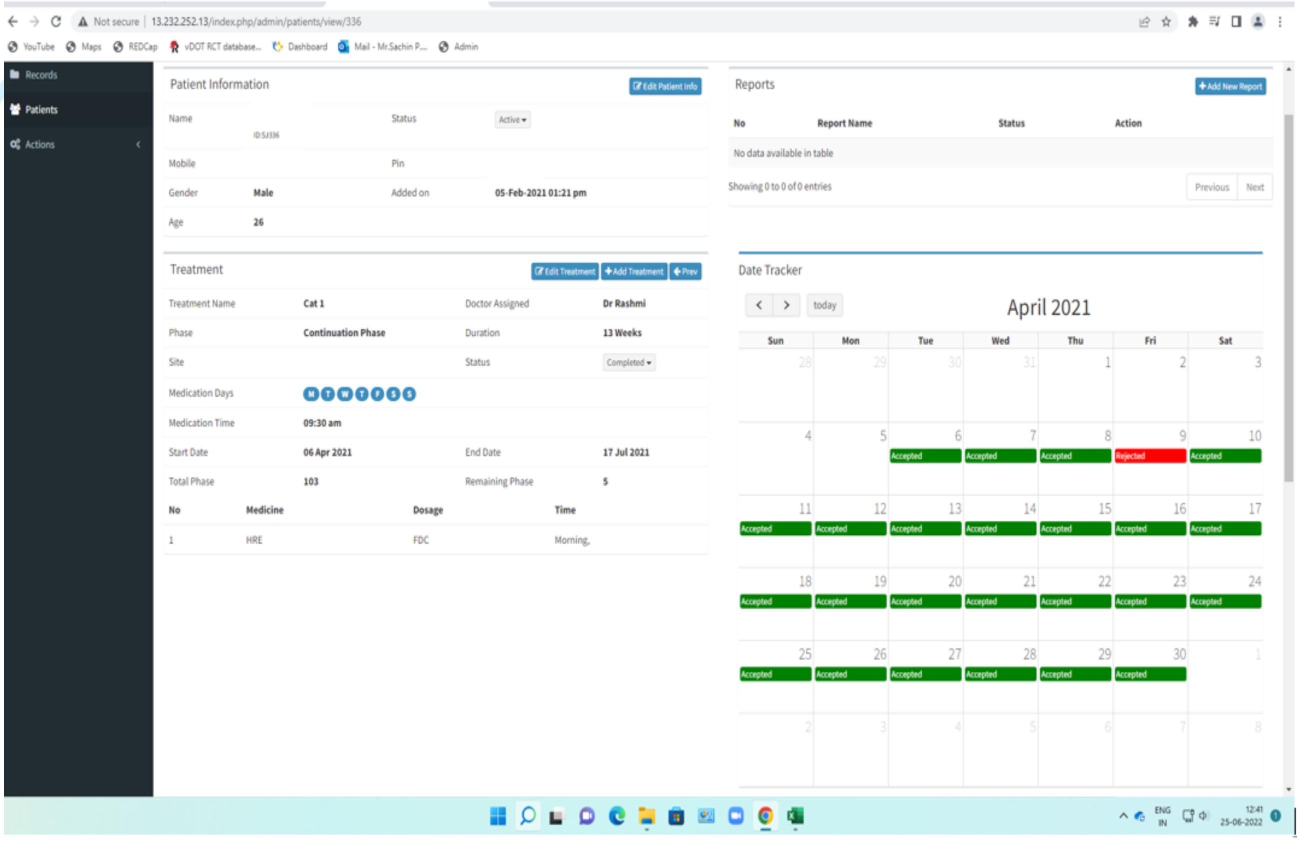

The patient record at the back – end of the Application.

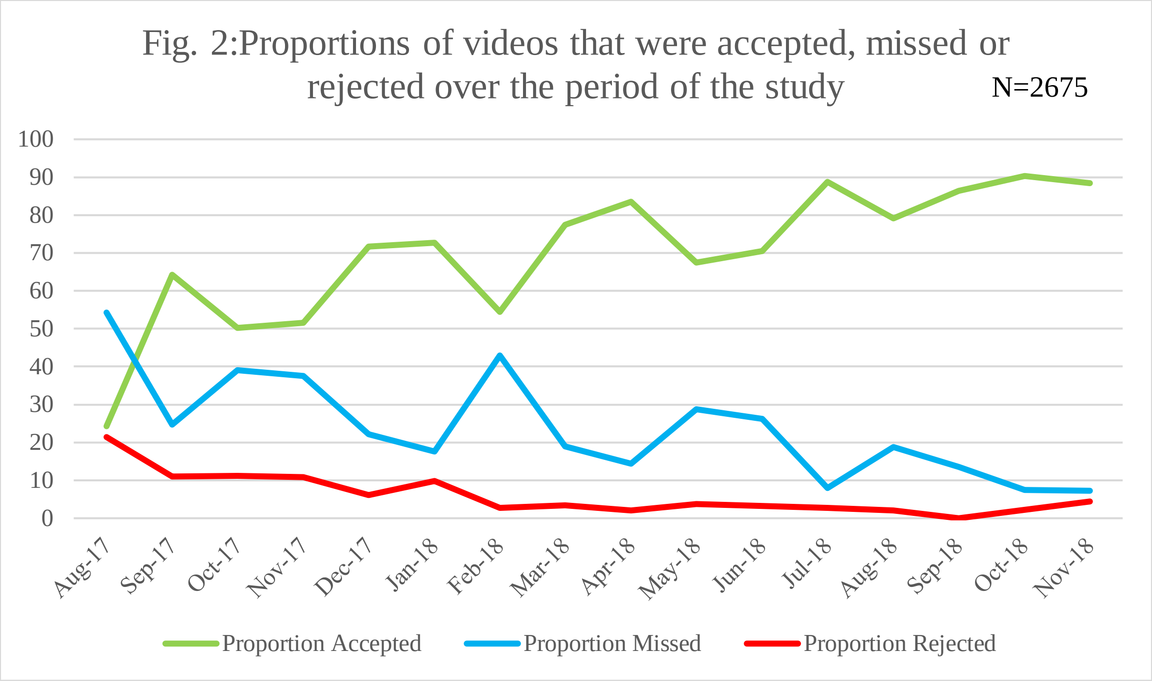

For peer review only

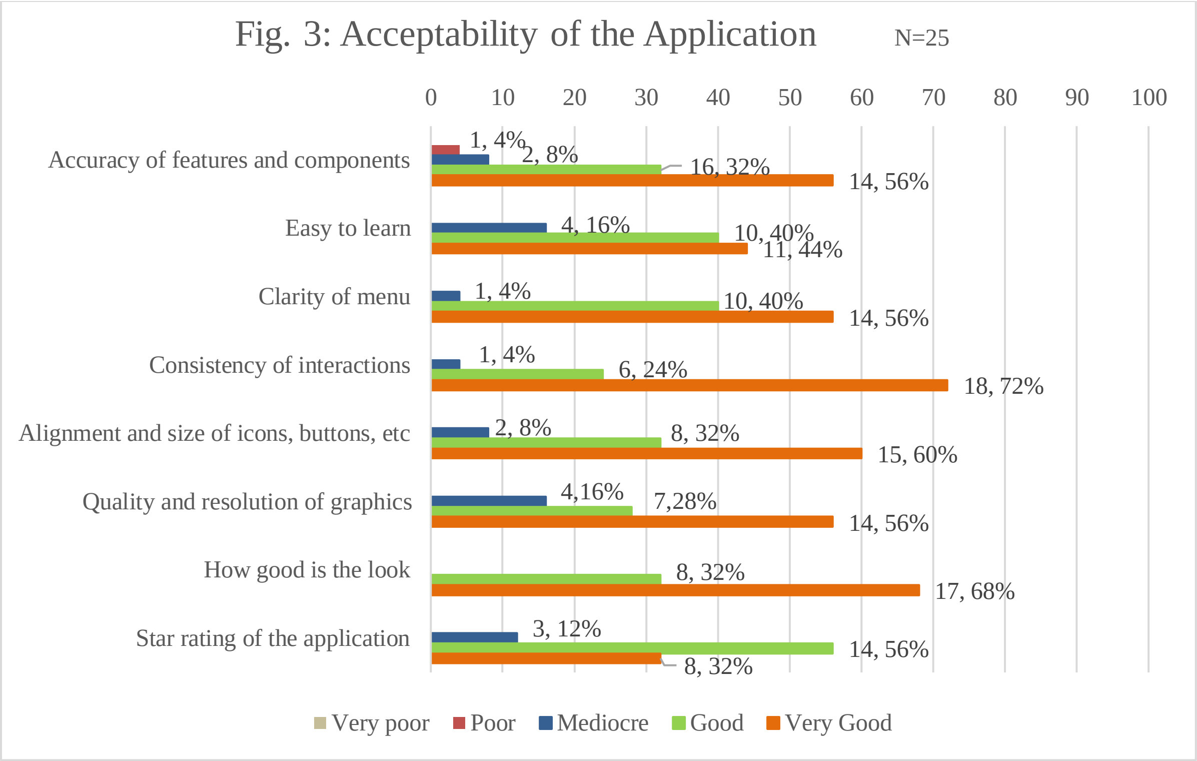
Fig. 3: Acceptability of the Application
N=25
0
10
20
30
40
50
60
70
80
90
100
Accuracy of features and components
1, 4%
2, 8%
16, 32%
14, 56%
Easy to learn
4, 16%
10, 40%
11, 44%
Clarity of menu
1, 4%
10, 40%
14, 56%
Consistency of interactions
1, 4%
6, 24%
18, 72%
Alignment and size of icons, buttons, etc
2, 8%
8, 32%
15, 60%
Quality and resolution of graphics
4,16%
7,28%
14, 56%
How good is the look
8, 32%
17, 68%
Star rating of the application
3, 12%
14, 56%
8, 32%
Very poor
Poor
Mediocre
Good
Very Good

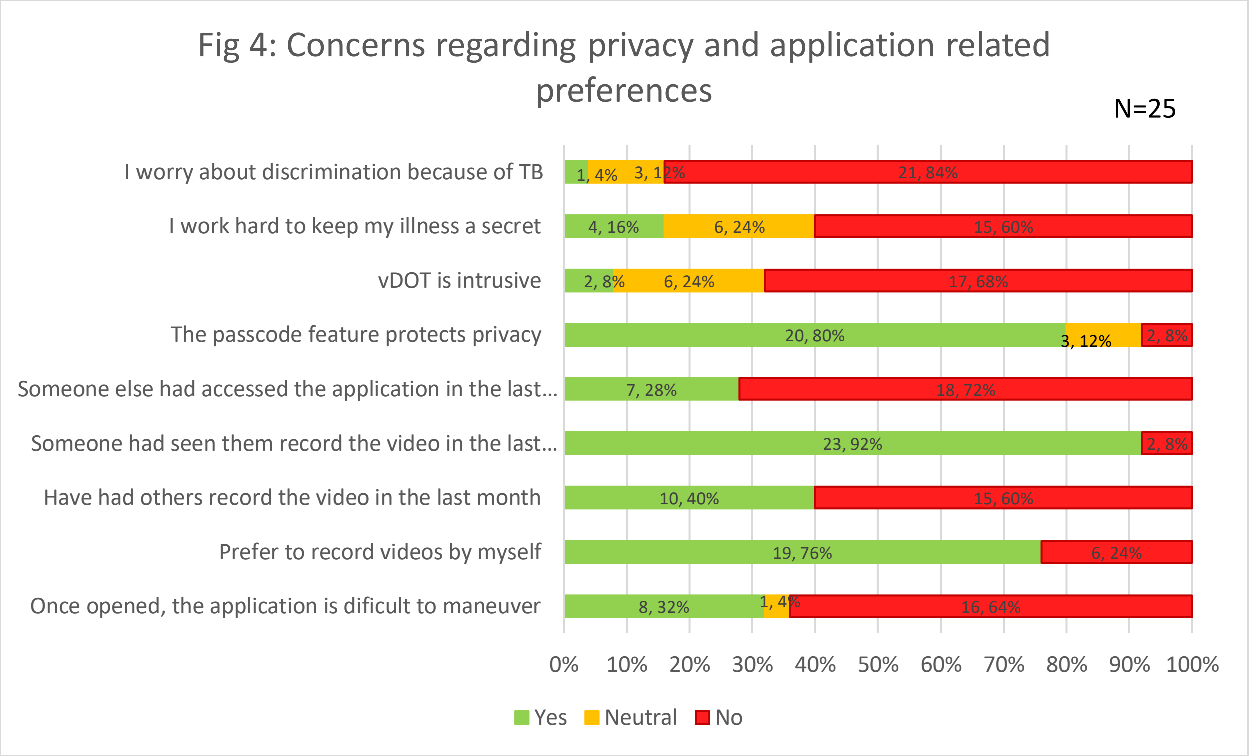

For peer review only

STROBE 2007 (v4) checklist of items to be included in reports of observational studies in epidemiology*
Checklist for cohort, case-control, and cross-sectional studies (combined)

Section/Topic	Item #	Recommendation	Reported on page #
**Title and abstract**	1	(*a*) Indicate the study's design with a commonly used term in the title or the abstract	1
		(*b*) Provide in the abstract an informative and balanced summary of what was done and what was found	2
**Introduction**			
Background/rationale	2	Explain the scientific background and rationale for the investigation being reported	3
Objectives	3	State specific objectives, including any pre-specified hypotheses	3
**Methods**			
Study design	4	Present key elements of study design early in the paper	4
Setting	5	Describe the setting, locations, and relevant dates, including periods of recruitment, exposure, follow-up, and data collection	4
Participants	6	(*a*) *Cohort study*—Give the eligibility criteria, and the sources and methods of selection of participants. Describe methods of follow-up<br>*Case-control study*—Give the eligibility criteria, and the sources and methods of case ascertainment and control selection. Give the rationale for the choice of cases and controls<br>*Cross-sectional study*—Give the eligibility criteria, and the sources and methods of selection of participants	5
		(*b*) *Cohort study*—For matched studies, give matching criteria and number of exposed and unexposed<br>*Case-control study*—For matched studies, give matching criteria and the number of controls per case	NA
Variables	7	Clearly define all outcomes, exposures, predictors, potential confounders, and effect modifiers. Give diagnostic criteria, if applicable	5
Data sources/ measurement	8*	For each variable of interest, give sources of data and details of methods of assessment (measurement). Describe comparability of assessment methods if there is more than one group	5
Bias	9	Describe any efforts to address potential sources of bias	5
Study size	10	Explain how the study size was arrived at	NA
Quantitative variables	11	Explain how quantitative variables were handled in the analyses. If applicable, describe which groupings were chosen and why	NA
Statistical methods	12	(*a*) Describe all statistical methods, including those used to control for confounding	5
		(*b*) Describe any methods used to examine subgroups and interactions	5
		(*c*) Explain how missing data were addressed	NA
		(*d*) *Cohort study*—If applicable, explain how loss to follow-up was addressed<br>*Case-control study*—If applicable, explain how matching of cases and controls was addressed	NA

For peer review only

		Cross-sectional study—If applicable, describe analytical methods taking account of sampling strategy	
		(*e*) Describe any sensitivity analyses	NA
**Results**			
Participants	13*	(a) Report numbers of individuals at each stage of study—eg numbers potentially eligible, examined for eligibility, confirmed eligible, included in the study, completing follow-up, and analysed	6
		(b) Give reasons for non-participation at each stage	NA
		(c) Consider use of a flow diagram	NA
Descriptive data	14*	(a) Give characteristics of study participants (eg demographic, clinical, social) and information on exposures and potential confounders	7
		(b) Indicate number of participants with missing data for each variable of interest	7
		(c) *Cohort study*—Summarise follow-up time (eg, average and total amount)	6 and 8
Outcome data	15*	*Cohort study*—Report numbers of outcome events or summary measures over time	8
		Case-control study—Report numbers in each exposure category, or summary measures of exposure	NA
		Cross-sectional study—Report numbers of outcome events or summary measures	NA
Main results	16	(*a*) Give unadjusted estimates and, if applicable, confounder-adjusted estimates and their precision (eg, 95% confidence interval). Make clear which confounders were adjusted for and why they were included	8
		(*b*) Report category boundaries when continuous variables were categorized	8
		(*c*) If relevant, consider translating estimates of relative risk into absolute risk for a meaningful time period	NA
Other analyses	17	Report other analyses done—eg analyses of subgroups and interactions, and sensitivity analyses	NA
**Discussion**			
Key results	18	Summarise key results with reference to study objectives	11
Limitations	19	Discuss limitations of the study, taking into account sources of potential bias or imprecision. Discuss both direction and magnitude of any potential bias	11
Interpretation	20	Give a cautious overall interpretation of results considering objectives, limitations, multiplicity of analyses, results from similar studies, and other relevant evidence	11
Generalisability	21	Discuss the generalisability (external validity) of the study results	11
**Other information**			
Funding	22	Give the source of funding and the role of the funders for the present study and, if applicable, for the original study on which the present article is based	12

*Give information separately for cases and controls in case-control studies and, if applicable, for exposed and unexposed groups in cohort and cross-sectional studies.

Note: An Explanation and Elaboration article discusses each checklist item and gives methodological background and published examples of transparent reporting. The STROBE checklist is best used in conjunction with this article (freely available on the Web sites of PLoS Medicine at http://www.plosmedicine.org/, Annals of Internal Medicine at http://www.annals.org/, and Epidemiology at http://www.epidem.com/). Information on the STROBE Initiative is available at www.strobe-statement.org.

For peer review only

BMJ Open

Feasibility and acceptability pilot of Video–based Direct Observed Treatment (vDOT) for supporting antitubercular treatment in South India – A cohort study

Journal:	*BMJ Open*
Manuscript ID	bmjopen-2022-065878.R1
Article Type:	Original research
Date Submitted by the Author:	21-Feb-2023
Complete List of Authors:	Rodrigues, Rashmi; St John's National Academy of Health Sciences, Community Health; Karolinska Institute, Public Health<br>Varghese, Suman; St John's National Academy of Health Sciences, St. John's Research Institute<br>Mahrous, Mohammed; Independent Researcher<br>Ananthaneni, Anil; St John's National Academy of Health Sciences, St. John's Research Institute<br>Ahmed, Mohammed; St John's National Academy of Health Sciences, St. John's Research Institute; Blackpool Teaching Hospital, NHS trust<br>D'Souza, George; St John's National Academy of Health Sciences, Pulmonary Medicine
**Primary Subject Heading**:	Epidemiology
Secondary Subject Heading:	Patient-centred medicine, Public health
Keywords:	Public health < INFECTIOUS DISEASES, INTERNAL MEDICINE, Epidemiology < INFECTIOUS DISEASES

SCHOLARONE™ Manuscripts

I, the Submitting Author has the right to grant and does grant on behalf of all authors of the Work (as defined in the below author licence), an exclusive licence and/or a non-exclusive licence for contributions from authors who are: i) UK Crown employees; ii) where BMJ has agreed a CC-BY licence shall apply, and/or iii) in accordance with the terms applicable for US Federal Government officers or employees acting as part of their official duties; on a worldwide, perpetual, irrevocable, royalty-free basis to BMJ Publishing Group Ltd ("BMJ") its licensees and where the relevant Journal is co-owned by BMJ to the co-owners of the Journal, to publish the Work in this journal and any other BMJ products and to exploit all rights, as set out in our licence.

The Submitting Author accepts and understands that any supply made under these terms is made by BMJ to the Submitting Author unless you are acting as an employee on behalf of your employer or a postgraduate student of an affiliated institution which is paying any applicable article publishing charge ("APC") for Open Access articles. Where the Submitting Author wishes to make the Work available on an Open Access basis (and intends to pay the relevant APC), the terms of reuse of such Open Access shall be governed by a Creative Commons licence – details of these licences and which Creative Commons licence will apply to this Work are set out in our licence referred to above.

Other than as permitted in any relevant BMJ Author's Self Archiving Policies, I confirm this Work has not been accepted for publication elsewhere, is not being considered for publication elsewhere and does not duplicate material already published. I confirm all authors consent to publication of this Work and authorise the granting of this licence.

For peer review only

Title: Feasibility and acceptability pilot of Video–based Direct Observed Treatment (vDOT) for supporting antitubercular treatment in South India – A cohort study

Authors: Rashmi Rodrigues[1,2,3](rashmijr@gmail.com), Suman Sarah Varghese[4] (sumansarah.92@gmail.com), Mohammed Mahrous[5] (mohammed.mahrousdr@gmail.com), Anil Ananthaneni[4] (anil_kumarans@yahoo.co.in), Mohammed Naseer Ahmed[4,6] (dr.m.naseer.ahmed@gmail.com), George A. D'Souza[7] (dsouza.ga1975@gmail.com)

1. Department of Community Health, St. John's Medical College, Bangalore, Karnataka, India
2. Department of Global Public Health, Karolinska Institutet, Stockholm, 10435, Sweden
3. DBT/Wellcome Trust India Alliance, Hyderabad/New Delhi, India
4. St. John's Research Institute, Bangalore, Karnataka, India
5. Independent researcher
6. Blackpool Teaching Hospital, NHS trust
7. Department of Pulmonary Medicine, St. John's Medical College, Bangalore, Karnataka, India

Corresponding Author:

Dr. Rashmi Rodrigues
Associate Professor, Department of Community Health,
St. John's Medical College, Bangalore -560034
Email id: rashmijr@gmail.com
Phone number: 9845389538

Word Count: 6606

For peer review only

ABSTRACT

Objectives: The objective of this study was to assess the feasibility and acceptability of video–based anti-Tuberculosis treatment adherence support in patients with TB in South India.

Design: An exploratory cohort

Setting: Participants were recruited at the Tuberculosis (TB) treatment center (DOTS center) of a tertiary level teaching facility in Bangalore, Karnataka, South India.

Participants: The study enrolled 25 patients with TB (PwTB), with replacement. Adult PwTB who were on drug sensitive treatment regimens were included, while those who had drug resistant TB were excluded from the study.

Intervention: Participants received scheduled adherence reminders and were trained to video-record themselves swallowing their medication via a mobile application. The application was automated to submit these videos for evaluation. Participants were followed-up monthly till treatment completion or withdrawal.

Outcome Measures: Adherence rate and acceptability of vDOT.

Results: The mean ± SD age of the participants was 33 ±14 years, majority were females (16, 64%), residing in urban areas (24,96%), married (17, 68%) and had access to smart phones (23,92%).A total of 3193 person days of follow – up was completed; of the videos submitted within the first six months of enrollment (2501), 94% (2354/2501) were considered 'acceptable' and sixteen (64%) participants were optimally adherent (i.e., ≥80%). Participant videos improved in quality and a higher proportion met acceptability criteria over time. Twenty-one (84%) participants stated that they found the application easy to learn; 13 (52%) preferred vDOT over DOT. Mixed model logistic regression showed that those who are married are more likely have daily adherence to ATT.

Conclusion: Video-based mobile phone interventions are acceptable to patients with TB and the ease of using the application increases with time. To provide patient centered care, vDOT is a promising option that can be offered to patients for treatment support and adherence monitoring.

Key words: mHealth, Pilot study, Treatment monitoring, Tuberculosis, video-based DOT

Strengths and limitations:

- Most study participants were women indicating that women are accepting of digital adherence technologies if given an opportunity.
- Videos not received were equated to missed doses, thereby underestimating treatment adherence, as participants may missed video recording an ingested dose.
- As this was a cohort study with a single group, Hawthorne effect may have played a role in increasing adherence under observation. However, the purpose of vDOT is 'observation', given the varied implementation of DOT.
- As this was an exploratory pilot, no sample size was calculated, limiting its power, the results of the study should be seen in this light. Yet the results provide valuable information regarding the feasibility and acceptability of vDOT.

For peer review only

Introduction:

Globally, 6.4 million people were diagnosed with tuberculosis in 2021 with India, contributing to approximately a third of these cases. (1). This, despite the availability of Anti-Tuberculosis Treatment (ATT), comprising antimicrobials and evidence based preventive strategies (2).

India has an annual TB incidence of 164 per 100000 population, of which 87% are newly diagnosed, 10% previously treated, and 2.8% Multi–Drug Resistant (MDR) patients with TB (PwTB). As per Indian TB guidelines ATT generally lasts six months and approximately 80% of PwTB are successfully treated annually (3). Further, 10% of PwTB are categorized as lost to follow-up (3), nonadherence to ATT, including loss to follow-up is the leading cause for the emergence of drug resistance – impacting costs of care, treatment duration and outcome (4). Further, Tuberculosis (TB) is distinctive as it impacts the working population and has sizeable economic ramifications (2).

In order to address the challenges with adherence to ATT, Direct Observed Treatment Short–course (DOTS) was adopted globally as a feasible solution in 1994 (5). Direct Observed Treatment (DOT), the fulcrum of the DOTS strategy, provides the much-needed follow–up as well as definitive evidence that medications were taken in the prescribed dose and manner. DOT, however, is not devoid of challenges. Facility based DOT requires patients to visit the treatment provider and often results in lost time and wages. On the other hand, community-based DOT runs the risk of compromising confidentiality due to health workers frequently visiting patients, leading to stigma. Further, such a strategy is human resource intensive from a programme perspective. These concerns, and the current ubiquity of digital communication via mobile phones have spurred innovative alternatives to DOT (5). Especially as India's smartphone penetration is 93% (91% rural and 97%) (6).

The Digital Adherence Technology (DAT) currently available to support ATT range from simple Short Message Service (SMS) reminders and video based directly observed treatment (vDOT) to sophisticated technologies with ingestible sensors embedded within pills (7). Further, the Indian National TB Elimination Programme (NTEP), while adopting a patient centered approach in 2014 (8), also included mHealth solutions for adherence support. Some of these mHealth solutions are (i) the 'Nikshay Sampark' call–center which uses Interactive Voice Response (IVR) and SMS as adherence reminders (7), (ii) the 'real time Medication Event Reminder Monitor (rtMERM),' - an electronic pill box with pressure sensors, that detects the weight of the pills in the box adherence prompts to patients based on the weight of the pill box (9–12), (iii) the 'Patient Compliance toolkit', an mHealth application with a smart card, that delivers adherence support comprising medication reminders, TB health tips, follow - up calls, and financial incentives (7).

"99DOTS," is another mHealth adherence support strategy that the NTEP, rolled out for patients with HIV--TB coinfection in 2015. The strategy subsequently covered all PwTB in 2021 during the Covid 19 pandemic, when DOTS posed a logistic challenge (13). 99DOTS requires, patients to give a missed call to a toll-free phone number that is revealed when the pills are popped from the ATT blister pack (14). The strategy minimizes travel, ensures privacy and supports remote monitoring (15). The value these digital innovations for adherence support, was realized during the COVID 19 pandemic, when DOT was impossible and remote monitoring the only viable option.

However, while most of these strategies support treatment adherence, video-based DOT (vDOT) is the only strategy that provides definitive evidence that the patient has in fact consumed the medication in the prescribed manner. Given the ubiquity of mobile phones, adherence support for ATT via mobile phones, especially vDOT is a suitable alternative to DOT (12). In this context, our study provides valuable evidence on the feasibility and acceptability of a video–based treatment adherence monitoring system (vDOT) among patients with pulmonary tuberculosis on Anti-Tuberculosis Treatment (ATT) in South India.

For peer review only

Methods:

This cohort study piloted the video DOT adherence support intervention for ATT at a private, tertiary care, teaching hospital in Bangalore, South India. The participants for this study were enrolled consecutively from those PwTB who were referred to the DOTS center for treatment between July 2017 and November 2018. As this was an exploratory study no sample size was calculated. The participants were recruited comprised newly diagnosed patients with drug sensitive TB, aged ≥18 years, with at least 2 months of treatment pending at enrolment. Participants recruited had to be willing to upload videos of themselves swallowing their ATT via the vDOT mobile application daily. PwTB who were ≥60 years, with HIV co-infection, recurrent TB or drug resistant TB were excluded from the study. Recruitment was done with replacement. Owning a mobile phone was not a necessity as participants were provided a mobile phone for the purpose of the study.

Study setting

St. John's Medical College Hospital (SJMCH), the study site, is a private, not-for profit, tertiary level, teaching, healthcare facility. The hospital provides emergency, outpatient, inpatient, community outreach, and day care services through its 1350 bedded hospital and outpatient facility. It is equipped with a network of laboratories that provide TB diagnostics such as smear microscopy, mycobacterial culture, and nucleic acid amplification test (NAAT, GeneXpert) as well as radiography. Patients diagnosed with TB at the hospital are referred for treatment to the TB treatment center (DOTS centre) at the hospital itself. The DOTS center functions through a public private partnership with the NTEP. PwTB receive treatment from the NTEP at the DOTS centre gratis. Alternatively, PwTB at SJMCH can also avail treatment for a cost at the hospital's pharmacy.

Standard Care

PwTB at SJMCH are treated in accordance with Indian TB treatment guidelines. Patients with drug sensitive TB receive daily treatment based on the their weight. The treatment comprises 2 months of an Intensive Phase (IP) with 4 drugs [Isoniazide (H), Rifampicin (R), Pyrazinamide (Z), Ethambutol (E)] followed by 4 months of a Continuation Phase (CP) with 3 drugs (H,R,E). This treatment is available in fixed dose combinations (FDC).

Conventionally, for ATT adherence support, a PwTB are expected to visit a health care provider 6 days a week and swallow their medications under direct supervision, but may be practiced variably. These visits are documented on a treatment card. Each PwTB receives a minimum of 180 days of treatment. If a dose (day) is missed, the treatment continues till the required number of doses are completed. While, adherence monitoring at the time of the study, was via in-person DOTS, currently, all PwTB receiving ATT through the NTEP receive 99DOTS.

The intervention: Video DOT (vDOT)

vDOT or video based Direct Observed Treatment, PwTB require to video–record themselves taking their medications via a mobile application, which then sends the videos to the study team via the internet. All participants also receive an adherence text reminder via the application daily at the time of their choice. For the purpose of this study the study team developed a vDOT mobile application the v.Cure, installed on the participants' mobile phones.

Trained research assistants (not routine health staff) reviewed the uploaded videos at the provider interface of the application, and classify the videos as '*accepted'* (dose taken) or '*rejected' (video received but dose not taken)*. The videos were accepted if the participant was identified, all the pills were shown before swallowing under adequate lighting. If not, the video was *'rejected'*. A participant is considered to have *"missed"* videos and thereby their ATT dose if no video was received for the day. Participants who missed uploading videos were contacted via their mobile phone on the subsequent day by the study team.

For peer review only

The vDOT mobile Application: v. Cure

The application comprised two intrefaces; (i) the user interface and (ii) the provider interface (fig 1)

User interface: The v.Cure, is available at the Google Play Store in five languages, i.e., English, Kannada, Hindi, Tamil and Telugu, each in their own script (https://play.google.com/store/apps/details?id=in.stjhons). The application is restricted to users in India. Only those registered by the healthcare provider can use the application. Once registered, the participant may log into the application installed on their mobile phones, using their credentials. The participant then views a instructional video within the application that demonstrates how to capture and upload the adherence video. Participants are free to skip the instructional video and access the recording screen directly to record the video. After the recording, the participant is informed of the status of the video, i.e., pending/uploaded. This is an automated process. The participant may choose the language for the application as well as the time for their adherence reminder notification at registration.

Provider interface: The provider interface has the demographic and clinical profiles of the participants, as well as their submitted video linked to their adherence calendar. The research staff view the video and indicate if the video is accepted or rejected, while the application itself flags a missed video. The adherence calendar reflects the status of the videos (accepted, rejected, missed), and also communicates the status to the participant via the v.Cure application.

Participant Enrolment and Follow-up

At baseline, trained research assistants administered consent, obtained relevant demographic and clinical information and installed the v.Cure mobile application onto the participants mobile phone. If the participant did not own a mobile phone but wished to participate in the study, a mobile phone was provided. The participant was then trained to use the mobile application and upload videos of themselves taking their medication. In case a participant as unable to use the application, a family member was chosen and trained to record and upload videos of the participant.

Study follow-ups were monthly and combined with pill refill visits, pill counts and clinical assessments. The v.Cure adherence calendar helped monitor adherence and reasons for missed videos were obtained via phone calls or at follow-ups. Acceptability of the intervention was assessed at the end of the study. Each participant received INR 500/month for internet, time, as well as study related travel.

Study outcomes:

Daily adherence at individual participant level: If a video is accepted the participant is considered adherent, while if missed or rejected participant is considered nonadherent.

The 'adherence rate' is the proportion of accepted videos to the total number of videos expected (includes accepted, rejected and missed). 'Optimal adherence' for the purpose of this study is defined as an adherence rate $\geq$ 80% (16).

Intervention Fidelity

All the participants who fulfilled the selection criteria and consented to participate in the study were trained in the use of vDOT application – registration, logging in, recording and uploading videos. Participant concerns regarding these procedures were addressed at registration and throughout the study duration. Further, phone numbers of study personnel were provided to participants to call in the event of a challenge with the application. The research team ensured that all the videos that were uploaded daily were viewed promptly. Participants who missed videos or submitted erroneous videos were contacted and their concerns recorded and addressed. Issues with application identified on the go were communicated with the technical team and addressed swiftly.

For peer review only

Analysis

Data comprising participant demography and clinical features obtained at baseline and follow-up were entered in Microsoft Excel. These data were combined with the adherence from the v.Cure application, also in Microsoft Excel. STATA v12.0 was used for analysis and the data described as frequencies and measures of central tendency and deviation. Participant experiences with the vDOT intervention were similarly described.

A multilevel mixed models logistic regression analysis was done with daily treatment adherence as the dependent variable. The fixed effects model includes participant demography and clinical factors as independent variables. The random effects model has two levels, the first was daily adherence, and at the second the individual participant (Fig 2). Adjusted Odds Ratios (95% Confidence interval), Intra-Class Cluster (ICC) coefficients and Akaike Information Criterion (AIC) are reported.

In addition, a mixed models logistic regression where the binomial dependent variable was daily participant responsiveness. Responsive meant video accepted or rejected or participant responded to follow-up call, nonresponsive meant participant did not respond to a follow-up call (Fig. 2; Table 1 annexure).

Patient and Public involvement

This was a part of a larger trial that uses vDOT as an adherence monitoring and treatment support tool (CTRI no: CTRI/2017/07/009052). Prior to the development of the intervention, acceptability of such an intervention among PwTB was conducted in Bangalore, South India and in Ujjain, Central India. (17,18). Patients' suggestions and concerns were taken into consideration during the design of the trial and during the development of the v.Cure application.

Results:

A total of 258 patients were screened, of whom 150 were eligible, and 40 were initially enrolled in the study. Eligible male participants (62, 56%) who refused consent cited 'time constraints' while eligible female participants (48,44%) cited privacy and stigma related concerns as reasons for refusal. The median time to enrollment from treatment initiation was 1 day (IQR 0-15 days) with a maximum of 109 days. The most common reasons for ineligibility were age <18 years or >60years (40, 37%), patients with HIV-TB coinfection (23, 21%) and recurrent TB (2 anti-tubercular therapy) (19, 17%).

Not owning/not interested in learning to use a smartphone [37(34%)] and not interested in research [20(18%)] were the most common reasons for declining to participate in the study (Table 1).

Of the 40 participants enrolled, 15 participants dropped out within two months of enrollment and were replaced; the most common reason for withdrawal was concern regarding privacy and stigma (5, 33%). The remaining 25 participants contributed 3193 person days of follow-up to the study.

Table 1: Reasons for replacing participants after enrollment and for refusing consent

**Sl. No.**	**Reasons cited**	**Number (%)**
Unwilling to participate		**N=110**
1	Not willing to learn to use a smartphone	37 (34%)
2	Not interested in research	20 (18%)
2	Stigma and Privacy Issues	22 (20%)
3	Time constraints	14 (13%)
4	Other Health Issues	6 (5%)
5	Family not comfortable	4 (4%)
6	Do not believe they have TB	3 (3%)
7	Would need to depend on others	2 (2%)
8	Network issues at home	2 (2%)

For peer review only

Participants who were replaced		**N=15**
1	Withdrew consent after participants' families were concerned of stigma and privacy	5 (33%)
2	Participants were unable to upload videos	3 (20%)
3	Participants were unable to find a family member to either take videos or upload them	3 (20%)
4	Lost to follow – up before two months of initiation of the intervention	3 (20%)
5	Network issues	1 (7%)

Demographic characteristics of the 25 study participants who completed at least 2 months of the intervention are given in the Table 2. Sixteen (64%) participants were female and 9 (36%) were male. Most participants had an urban background (96%), were married (68%), and had access to a smartphone (92%). The median income of the participants was □15,000 (IQR 7,000 – 22,750). Nearly equal numbers of participants preferred vDOT (52%) to DOT (48%).

For peer review only

Table 2: Socio–demographic and clinical features

Factor<br>N	Category	Female<br>16	Male<br>9	Total<br>25
Age, mean ±SD		32.8 ± 15	33.6 ± 12	33 ± 14
Family income*, n (%)	BPL	6 (38%)	7 (78%)	13 (52%)
	APL	10 (62%)	2 (22%)	12 (48%)
Residence, n (%)	Rural	0 (0%)	1 (11%)	1 (4%)
	Urban	16 (100%)	8 (89%)	24 (96%)
Marital status, n (%)	Single	6 (38%)	2 (22%)	8 (32%)
	Married	10 (62%)	7 (78%)	17 (68%)
Children, n (%)	Yes	9 (56%)	6 (67%)	15 (60%)
	No	7 (44%)	3 (33%)	10 (40%)
Owns a phone, n (%)	Yes	15 (94%)	9 (100%)	24 (96%)
	No	1 (6%)	0 (0%)	1 (4%)
Access to a Smartphone, n (%)	Yes	14 (87%)	9 (100%)	23 (92%)
	No	2 (13%)	0 (0%)	2 (8%)
TB site, n (%)	Pulmonary	4 (25%)	6 (67%)	10 (40%)
	Extra Pulmonary	12 (75%)	3 (33%)	15 (60%)
Median duration between treatment initiation and enrolment (in days)		1.5 (0 – 12.25)	1 (0 - 9)	1 (0 - 15)
Phase of treatment at the time of enrolment	Intensive Phase	14 (87%)	8 (89%)	22 (88%)
	Continuation Phase	2 (13%)	1 (11%)	3 (12%)
Prefer vDOT	Yes	10 (62%)	3 (33%)	13 (52%)
	No	6 (38%)	6 (67%)	12 (48%)
Remained till treatment completed treatment, n (%)	No	3 (19%)	0 (0%)	3 (12%)
	Yes	13 (81%)	9 (100%)	22 (88%)

#Withdrew consent: 3, 2nd month 2 (due to family concerns regarding stigma), 3rd month 1 (due to other health related issues); *according to WHO standards, BPL- Below Poverty Line, APL – Above Poverty Line

For peer review only - http://bmjopen.bmj.com/site/about/guidelines.xhtml

Adherence via vDOT:

vDOT adherence assessment required a minimum of 2 months and a maximum of six months within the intervention. During this period in the study we received 78% (2501/3193) of the expected videos from the participants. Of the videos received, 94% (2354/2501) were 'accepted'. The proportion of uploaded videos that met the acceptability criteria increased over time as participants' familiarity with the application grew. (Fig. 3).

The overall average adherence was 70% (95%CI: 68%-87%), and ranged between 12-99%. Sixteen (64%) participants were optimally adherent through vDOT of ≥ 80% [mean adherence (95% CI) 91% (88%-95%)]. The remainder had less than 80% of videos accepted [12 participants, mean (95% CI) adherence 51% (38% - 68%)].

Participants who were female, those married, those literate, those gainfully employed, those below poverty line socio-economic status, those who were enrolled earlier on in their treatment and those who preferred DOT were more adherent to medication (Table 3). However, the analysis lacks power.

Table 3: Comparison between proportion optimally adherent and demography

**Factors**	**Optimally adherent (n)**	**Total N=25**	**Unadjusted OR (95% CI)**
**Sex**			
**Female**	10 (63%)	16	1.20 (0.16 – 12.3)
Male	06/16 (67%)	09	*Reference*
**Age [mean ±SD] yrs (n=16)**	34 ±16	33 ±14	-
**Employment**			
Unemployed	07 (64%)	11	0.63 (0.09 – 4.27)
Employed	09 (64%)	14	*Reference*
**Socio-economic Status**			
**BPL**	09 (64%)	14	1.03 (0.14 – 7.04)
APL	07 (64%)	11	*Reference*
**Literacy**			
Illiterate	02 (67%)	03	0.88 (0.01 –19.52)
**Literate**	14 (64%)	22	*Reference*
**Marital Status**			
Unmarried	06 (75%)	08	0.49 (0.04 – 3.94)
**Married**	10 (59%)	17	*Reference*
Time to treatment initiation in days (mean±SD) n=16	1.19 ± 0.4	1.75 ± 0.33	-
**Participant preference**			
**vDOT**	08(62%)	13	1.24 (0.18 – 8.87)
DOT	08(67%)	12	*Reference*

A mixed model analysis was carried out with daily adherence to ATT as the outcome variable. The ICC of the null model indicated that 33% of the variation was contributed by inter cluster variation. The final model showed that those who were unmarried were more likely to have daily adherent (Table 4).

For peer review only

Table 4: Mixed-effects multilevel logistic regression analysis of the determinants of daily adherence to ATT

Variable		Null Model	Logistic regression	Final Model
Fixed Effects Model		Adjusted Odds Ratio (95% Confidence Interval)	Adjusted Odds Ratio (95% Confidence Interval)	Adjusted Odds Ratio (95% Confidence Interval)
Sex	Females		0.44 (0.31 – 0.62)	0.43 (0.06 – 1.99)
	Male		*Reference*	*Reference*
Age			0.99 (0.98 – 1.0)	0.98 (0.94 – 1.03)
Area of residence	Urban		3.86 (2.02 -7.38)	
	Rural		*Reference*	
Marital status	Married		0.41 (0.30-0.57)	0.42 (0.30-0.58)
	Unmarried		*Reference*	*Reference*
Employment status	Employed		0.18 (0.03 – 1.42)	0.18 (0.02 –1.43)
	Unemployed		*Reference*	*Reference*
Family income	Above Poverty Line		1.82 (1.5 – 2.22)	2.13 (0.70 – 6.52)
	Below Poverty Line		*Reference*	*Reference*
Literacy	Literate		0.32 (0.20 – 0.52)	0.35 (0.03 -3.65)
	Illiterate		*Reference*	*Reference*
Owns a phone	Yes		0.16 (0.99 -1.0)	0.13 (0.006 – 2.94)
	No		*Reference*	*Reference*
Site of TB	Pulmonary TB		0.98 (0.78 – 1.23)	1.07 (0.34 – 3.38)
	Extrapulmonary TB		*Reference*	*Reference*
Patient Preference of vDOT	Prefers vDOT over DOT		0.45 (0.35 – 0.58)	0.94 (0.23 – 3.77)
	Prefers DOT over vDOT		*Reference*	*Reference*
Time from treatment initiation to enrolment			1.0 (0.99 – 1.0)	0.99 (0.94 – 1.03)
Random Effects Model				
At individual level				1.09 (0.81 – 1.47)
Intra class cluster coefficient (95%CI) at Individual level		0.34 (0.22 - 0.48)		0.27 (0.17 – 0.40)
Akaike Information Criterion (AIC)		2966	3457	2978

All 25 participants had at least one missed video. Of the 692 missed instances, participants could not be contacted in nearly half (326) of these instances. A mixed model analysis was carried out with responsiveness as the outcome variable. The ICC for the null model indicates that 70% of the variation is accounted for by inter cluster variation. The model indicated that those who were unemployed were more likely to have daily responsiveness during the follow – up period (Annexure table 1).

For the 460 (66%) missed instances wherein participants responded to calls, 'technical issues' with the application was cited as the most common reason for not uploading the video (Table 5).

For peer review only

Table 5: Reasons for not uploading (missed) videos among those who were contacted

Sl. No.	Reasons	Instances, n=460	Participants N=25
1	Technical issues - application or phone	89 (19%)	15 (60%)
2	Claimed to have uploaded	87 (19%)	17 (68%)
3	Travel – personal or health related	76 (17%)	11 (44%)
4	Lost phone/ phone repair	33 (7%)	8 (32%)
5	Shared Phone/dependent	30 (7%)	4 (16%)
6	Forgot to upload Video	25 (5%)	10 (40%)
7	No Network	23 (5%)	10 (40%)
8	Wants to withdraw	19 (4%)	3 (12%)
9	No power	18 (4%)	10 (40%)
10	Forgot to take medicines	12 (2%)	3 (12%)
11	Change in schedule/phase	11 (2%)	5 (20%)
12	Still Learning the App	15 (3%)	4 (16%)
13	Accidentally deleted the app	8 (2%)	7 (28%)
14	In Person DOTS	7 (2%)	8 (32%)
15	No Permission from DOTS provider	7 (2%)	3 (12%)

Acceptability and feasibility of vDOT

Majority of the participants reported perfect functioning of the application. Eleven (44%) participants faced some challenges with accuracy and speed of the application features and components. Most participants stated that they were either able to use the application immediately [11(44%)] or it was easy to use [10(40%)]. Majority of the participants found the menu labels/icons/buttons to be perfectly clear [14(56%)]. Participants commonly expressed that interactions were perfectly consistent and intuitive across all components and screens (Fig. 4).

All the participants felt that the arrangement and size of the buttons/icons/menus/content on the screen were satisfactory and appropriate. Over half the participants reported that the resolution of the graphics used was of very high quality. Majority stated that the application had very high visual appeal (Fig. 4). Less than two – fifths of the participants found the application difficult to maneuver once opened.

Privacy and application related preferences

Most of the participants were not worried about discrimination due to TB (84%). While 2 (8%) participants found vDOT intrusive (8%), most participants found the passcode feature effective in protecting their privacy [20(80%)]. A quarter of the participants stated that the mobile application had been accessed by others in the preceding month and nearly all the participants reported that someone had seen them recording the video. However, none of these instances were followed by a negative experience. Most participants preferred recording the videos themselves. (Fig. 5)

Intervention Fidelity

The most common reason for a video not uploaded or recorded as uploaded but not received by the study team was “technical issues with the application” this referred to issues at both, the user and provider interface (89, 19%). These problems (table 6) were resolved after discussion with application developers. An interesting issue identified was incompatibility of the application with a few lower-end smart phone models. This was resolved by adjusting settings in the Google playstore, while mobile phone models that continued to be incompatible with the application were not used in the study. Participants with such phones or those without phones were given a smart phone for the purpose of participation.

Table 6: Issues identified with the vDOT application

For peer review only

Sl. No.	Issue raised
Participant perspective	
1	The pop – up reminders intended to prompt participants to take medications were not working
2	In case participants forgot their passcode to access the application, they would contact the team and a new pin would be generated.
Research team perspective	
3	The application was not compatible with two specific smartphone models
4	The application was designed to automatically calculate the 'acceptance ratio', however, the calculation was inaccurate
5	Once the video received from the participant was reviewed, the participant list shuffled
6	The provider interface screen would hang after rejecting a video
7	At times uploaded videos, could be viewed only after 1 –2 days
8	The missed instances were not sorted in any particular order, we requested the application developers to sort them by date
9	Missed doses were not captured in the data tracker calendar for individual participants
10	Percentage adherence, at times, exceeded 100%

Discussion

This study aimed to assess the acceptability and feasibility of vDOT for ATT adherence support. We observed that nearly two – thirds (16, 64%) of the participants had optimal adherence (i.e.,≥80%) with vDOT, with no sociodemographic factors influencing adherence. Most participants found the application easy to use, with high visual appeal. Those who belonged to above poverty live were more likely to have daily adherence to ATT.

While the most common reasons for not participating in the study were unwillingness to learn how to use a smartphone and disease related stigma, known barriers to mHealth are poor internet connectivity, security and privacy as well as English literacy, as reported in other studies, also played a role (19). However, as the application was video based it required participants to learn the skill of opening the application and recording the video. This does not require language literacy. Further, as the participants were largely urban, internet connectivity was not an issue.

Of the 2501 doses received on the application, over 90% were of acceptable quality, While 40% of the participants forgot to record a video at least once during follow-up, only 12% had actually missed taking their medication. A cross-sectional study done among HIV-TB co-infected patients using 99DOTS, reported that 26% had forgotten to take the medication and 6% participants forgot to make a call after taking the medication (20). This could be attributed to the fact that unlike 99DOT, where the patient gives a missed call after taking the medication, in vDOT the act of taking the medication is linked to recording the video thereby minimising missed instances.

However, for vDOT, if we did not receive a video we equated it to a missed dose. While doing so underestimated the true adherence it likely ensured robustness. This especially, as the viewing every dose taken via the mobile application, is the alternative to DOT that we propose. While this might imply vDOT as a standalone intervention is questionable, evidence indicates that DAT such as vDOT holds promise when combined with other interventions such as family/community DOTS or peer support.

Furthermore, mHealth interventions are expected to improve the quality, accessibility and cost – effectiveness of healthcare interventions available to patients (19). According to the National Strategic Plan for TB (NSP) 2017-2025 a good patient support plan is vital for treatment success and should be developed at the time of treatment initiation (7). Educating patients, families and communities regarding TB and its treatment while improving access to patient centered care also improves treatment adherence and outcomes (17). The vDOT application also has the potential to address these needs if tailored to the individual, thereby personalizing the intervention.

For peer review only

Being asynchronous, vDOT has similar benefits as 99DOTS and sensor observed treatment (SOT), namely, convenience, decreased absenteeism from work and reduction in travel costs (18). Additionally, logistic difficulties during the manufacturing and supply of customized sleeves for the ATT blister packs used in 99DOT are avoided (21). vDOT can also minimize the discomfort or extra effort associated with SOT, which requires the patient to wear wireless wearable readers that capture data from the sensor within the ingested pill. The wearable readers are usually worn on their wrist, arm, hip or abdomen, which if seen by others can potentially increase stigma. On the other hand vDOT is considered a less intrusive and a more autonomous adherence monitoring tool (19,20) as the participant can use an inconspicuous mobile phone for the purpose. Besides, patients may find ingesting a sensor intrusive (22) and the bulk of the wearable sensor is a barrier to its uptake (23-25).

The vDOT application we developed uses a passcode to authenticate access to the application. When participants forgot their passcode, they called the study team to generate a new code. Aside from concerns regarding the authenticity of the caller, this system worked efficiently to provide participants with timely access to the application. However, we did not receive any complaints from the participants stating that others had accessed their phones and generated passcodes. Further, as the passcodes as well as the user IDs were numeric, only digital literacy was required.

The limitation of the current version of the application is that it does not support the reporting of adverse events (AEs). The decision to exclude this feature was made by the research team to reduce complexity in terms of resources required. Albeit participants were provided with appropriate contact numbers to reach out to the study team in an emergency. In the future, the application may be modified to include reporting of AEs by patients. The vDOT minimized patient – provider interaction. To overcome this drawback, participants were called whenever a missed dose was recorded, in addition to the monthly visits to the facility.

Interventional fidelity was ensured by uncompromising adherence to the protocol, appropriate training of participants to use the application and timely redressal of challenges identified– which was one of the purposes of the pilot. While patient preferences may depend on familiarity with the mHealth delivery platform (26), our study did not find familiarity to influence adherence and is likely due to the small sample size. The study also shows that patients with no exposure to DAT can be successfully trained to use them, which was confirmed with the increasing proportion of videos that were accepted increases with time. Overall, while the small sample size limits generalisability, the study has helped identify and address technical challenges with the application and while understanding its acceptability. Also, approximately 2/3rd of the participants were women, as many men with TB refused to participate in the study citing employment and data security issues. This implies a selection bias as the proportion of men with TB is higher than that of women and so also their access to digital technologies (27). However, the valuable insight into the uptake of DAT by women, that the study provides, if given an opportunity, cannot be ignored.

Additionally, this was a single arm cohort study that not only excluded participants unwilling to comply with the protocol but also replaced some. While this makes the study more explanatory than pragmatic, it is also subject to the Hawthorne effect, which however, addresses the premise of vDOT i.e., improving treatment adherence through observation, as the implementation of DOT may vary.

Conclusion

We found that vDOT is an acceptable alternative to conventional DOT for ATT adherence support and monitoring. The application was easy to learn and nonintrusive. Though concerns with privacy and stigma limited participation, features like the passcode to access the mobile application helped alleviate participant concerns. However, given the increasing digital literacy and the growing confidence in digital technology in the backdrop of pandemics such as the Covid 19, the use of vDOT for adherence support in tuberculosis is

imperative. Yet, with the availability of multiple digital adherence interventions, PwTB may be offered a basket of choices– similar to the "cafeteria approach" for contraception, for treatment adherence support – of which vDOT is one.

Data Availability

The data is made available on open science framework (DOI 10.17605/OSF.IO/Z4S9C)

Data are available under the terms of the Creative Commons Attribution 4.0 International license (CC-BY 4.0).

Funding Statement

This work was supported by DBT/Wellcome Trust India Alliance (number IA/CPHI/15/1/502042) and the Swedish Research Council (number 2015-02750).

Acknowledgements

The authors would like to thank Ms Shubha Krishnamurthy, Ms Hannah DJ, Dr Nikitha Prabhakar, and Dr Naseer Ahmed who supported data collection and data management. The authors also appreciate the support from the offices of the district TB officers, Bruhat Bengaluru Mahanagara Palike, and Bangalore Urban District, Bangalore, India, through their respective public health care facilities in urban and rural Bangalore. We would like to thank Focaloid Technologies for their dedication and approachability that was instrumental in the development of the application.

Conflict of interest

The authors have no conflict of interest.

Author contribution

RR and GD were involved in the concept and design of the study and in monitoring the study, AAK and AN were involved in administering informed consent, data collection, entry and monitoring, MM and SSV were involve in drafting the paper and data analysis, RR was involved in the final revision of the article.

Ethics Statement

Ethics clearance was obtained from the Institutional Ethics Committee, St. John's Medical College, Bangalore (IEC no 342/2016). Written informed consent was obtained from all individuals willing to participate. Participants were provided study related information in a language that they understood. For illiterate participants, all information was imparted orally and thumb impression along with the signature of an impartial witness obtained.

References

1. Global Tuberculosis report 2022. Geneva: World Health Organisation. 2022.

2. Rajeshwari R, Balasubramanian R, Muniyandi M, Geetharamani S, Thersa X, Venkatesan P. Socio-economic impact of tuberculosis on patients and family in India Int J Tuberc Lung Dis [Internet]. 1999 Oct [cited 2022 Apr 5];3(10):869–77. Available from: https://www.ingentaconnect.com/content/iuatld/ijtld/1999/00000003/00000010/art00005

3. Ministry of Health and Family Welfare. INDIA TB REPORT 2022 - Coming Together to End TB Altogether [Internet]. New Delhi; 2022 [cited 2022 May 11]. Available from: https://tbcindia.gov.in/WriteReadData/IndiaTBReport2022/TBAnnaulReport2022.pdf

4. Central TB Division. India TB Report 2019. Welfare, Ministry of Health and Family. 2019. p. 244.

5. Chauhan LS, Tonsing J. Revised national TB control programme in India. Tuberculosis. 2005 Sep;85(5–6):271–6.

6. Mohan D, Bashingwa J, Tiffin N, Dhar D, Mulder N, George A, et al. Does having a mobile phone matter? Linking phone access among women to health in India: An exploratory analysis of the National Family Health Survey. PLoS One. 2020;15(7).

7. National Strategic Plan for Tuberculosis Elimination 2017-2025 [Internet]. 2017 [cited 2022 Apr 5]. Available from: https://tbcindia.gov.in/WriteReadData/NSP Draft 20.02.2017 1.pdf

8. Central TB Division Ministry of Health & Family Welfare Government of India and World Health Organization; Standards for TB care in india. 2014.

9. Liu X, Blaschke T, Thomas B, De Geest S, Jiang S, Gao Y, et al. Usability of a medication event reminder monitor system (MERM) by providers and patients to improve adherence in the management of tuberculosis. Int J Environ Res Public Health. 2017;14(10):1–11.

10. Wang N, Shewade HD, Thekkur P, Huang F, Yuan Y, Wang X, et al. Electronic medication monitor for people with tuberculosis: Implementation experience from thirty counties in China. PLoS One. 2020;15(4):1–14.

11. Hayes TL, Hunt JM, Adami A, Kaye JA. An Electronic Pillbox for Continuous Monitoring of Medication Adherence. Conf Proc IEEE Eng Med Biol Soc [Internet]. 2006;1:6400. Available from: /pmc/articles/PMC2911441/

12. Thomas BE, Kumar JV, Periyasamy M, Khandewale AS, Mercy JH, Raj EM, et al. Acceptability of the Medication Event Reminder Monitor for Promoting Adherence to Multidrug-Resistant Tuberculosis Therapy in Two Indian Cities: Qualitative Study of Patients and Health Care Providers. J Med Internet Res [Internet]. 2021 Jun 1;23(6). Available from: https://pubmed.ncbi.nlm.nih.gov/34110300/

13. Central TB Division. TB India 2017. 2017.

14. Government of India. Using Mobile phones to Improve Adherence to to Tuberculosis Medication. Central TB Division of India, Minsitry of Health and Family Welfare. 2021.

15. Oberoi S, Kumar Gupta V, Chaudhary N, Singh A. 99 DOTS.; Available from: www.ijcmr.com

16. Nahid P, Dorman SE, Alipanah N, Barry PM, Brozek JL, Cattamanchi A, et al. Official American Thoracic Society/Centers for Disease Control and Prevention/Infectious Diseases Society of America Clinical Practice Guidelines: Treatment of Drug-Susceptible Tuberculosis. Infect Dis Soc Am. 2016;63.

17. Kumar AA, De Costa A, Das A, Srinivasa GA, D'souza G, Rodrigues R. Mobile Health for Tuberculosis Management in South India: Is Video-Based Directly Observed Treatment an Acceptable Alternative? JMIR mHealth uHealth. 2019;7(4). Available from: /pmc/articles/PMC6468344/

18. Rao JS, Diwan V, Kumar AA, Varghese SS, Sharma U, Purohit M, et al. Acceptability of video observed treatment vs. directly observed treatment for tuberculosis: a comparative analysis between South and Central India. Wellcome Open Res 2022 7152. 2022 May 13;7:152. Available from: https://wellcomeopenresearch.org/articles/7-152

19. Wipro Council for Industry Research. The mHealth Case in India . Bangalore; 2013. Available from: www.wipro.com

20. Ghatage S, M S A, Aithal SS, D B S, Angadi N. A study on reasons for nonadherence to 99DOTS among HIV-tuberculosis coinfected patients in Davanagere district, Karnataka. Int J Med Sci Public Heal [Internet]. 2018;7(10):805–8. Available from: http://www.ijmsph.com/fulltext/67-1528277879.pdf?1641794950

For peer review only

21. Prabhu A, Agarwal U, Tripathy JP, Singla N, Sagili K, Thekkur P, et al. "99DOTS"techno-supervision for tuberculosis treatment – A boon or a bane? Exploring challenges in its implementation at a tertiary centre in Delhi, India. Indian J Tuberc. 2020 Jan 1;67(1):46–53.

22. Gebreweld FH, Kifle MM, Gebremicheal FE, Simel LL, Gezae MM, Ghebreyesus SS, et al. Factors influencing adherence to tuberculosis treatment in Asmara, Eritrea: A qualitative study. J Heal Popul Nutr. 2018;37(1):1–9.

23. Mason M, Cho Y, Rayo J, Gong Y, Harris M, Jiang Y. Technologies for Medication Adherence Monitoring and Technology Assessment Criteria: Narrative Review. JMIR mHealth uHealth [Internet]. 2022 Mar 1;10(3). Available from: /pmc/articles/PMC8949687/

24. Kumar K, Loebinger MR, Ghafur S. The role of wirelessly observed therapy in improving treatment adherence. Futur Healthc J. 2022;9(2).

25. Chai PR, Goodman G, Majo B, Mendez L, Yassir M, et al. Design and Delivery of Real-Time Adherence Data to Men Who Have Sex with Men Using Antiretroviral Pre-exposure Prophylaxis via an Ingestible Electronic Sensor. 2020; Available from: https://doi.org/10.1007/s10461-020-03082-y

26. Distefano MJ, Schmidt H. mHealth for Tuberculosis Treatment Adherence: A Framework to Guide Ethical Planning, Implementation, and Evaluation.; Available from: www.ghspjournal.org

27. Horton KC, MacPherson P, J. Houben MG, White RG, Corbett EL. Sex Differences in Tuberculosis Burden and Notifications in Low- and Middle-Income Countries: A Systematic Review and Meta-analysis. PLoS Med. 2016;13(9).

Figure Legend:

Figure 1: v.Cure Application operation

Figure 2: Schematic presentation of the levels in the hierarchical model

Figure 3: Proportions of videos that were accepted, missed or rejected over the period of the study

Figure 4: Acceptability of the Application

Figure 5: Concerns regarding privacy and application related preferences

For peer review only

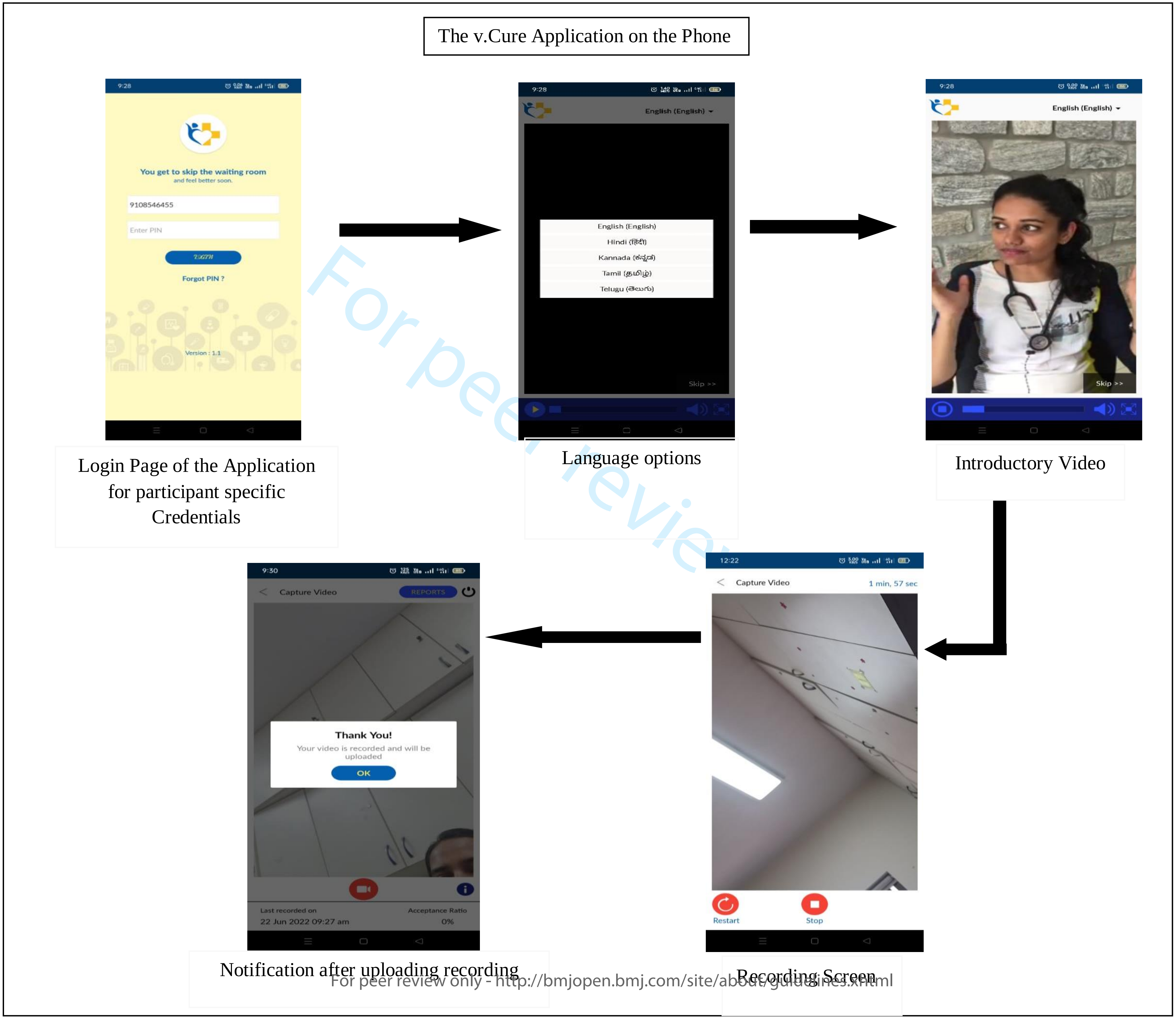

For peer review only - http://bmjopen.bmj.com/site/about/guidelines.xhtml

The v.Cure Application on the Back – end

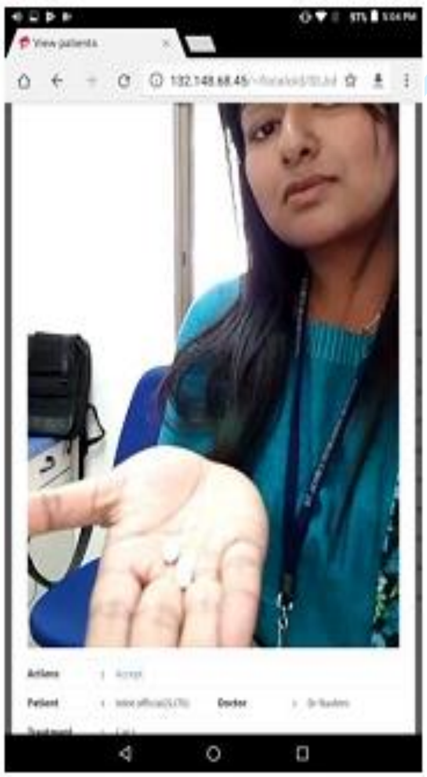

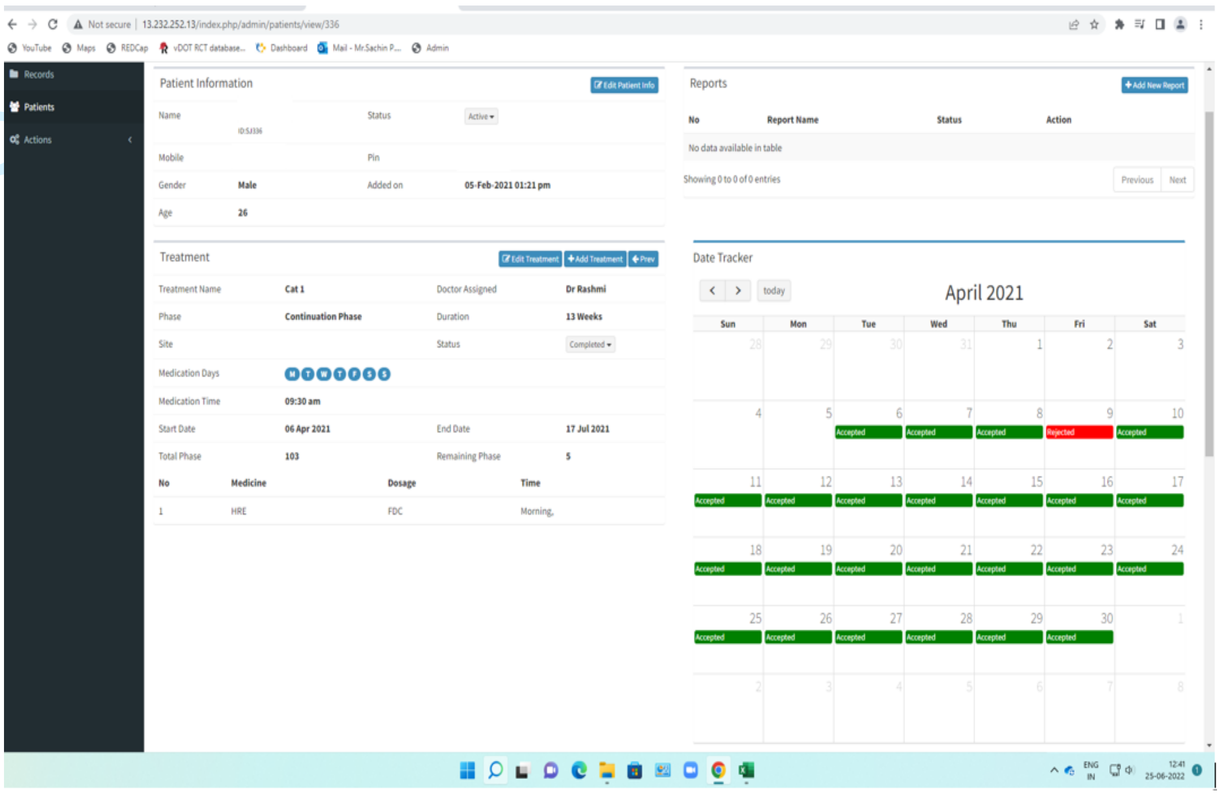

Video recording that is viewed on the back – end of the Application. Actionable: Reject/Accept

The patient record at the back – end of the Application.

Figure 2: Schematic presentation of the levels in the hierarchical model

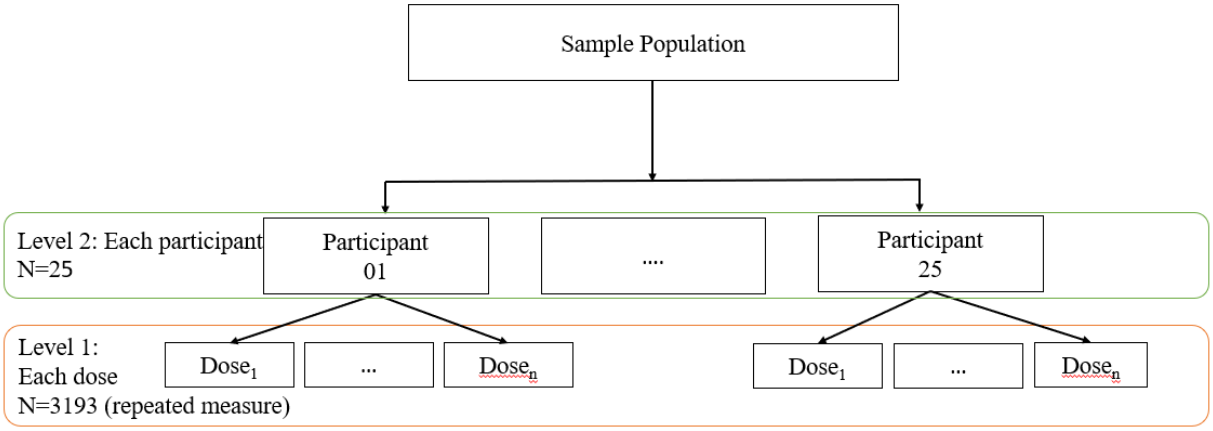

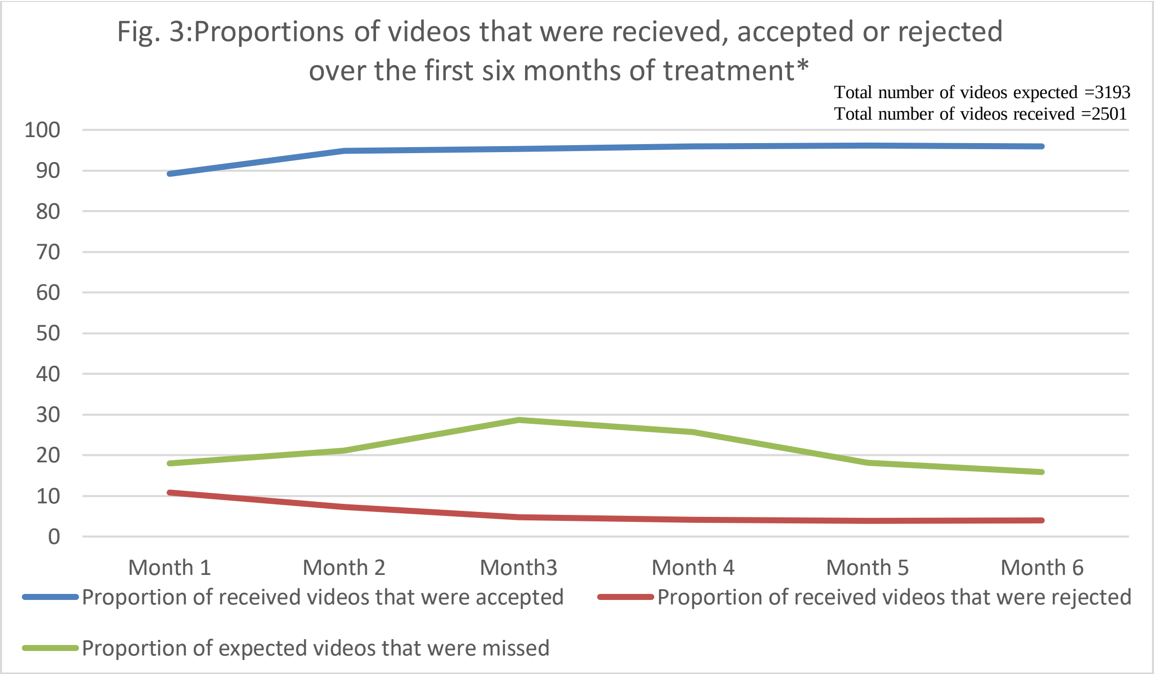

*three participants had treatments that exceeded 6 months, however, the values in this graph are restricted to the first six months of treatment only.

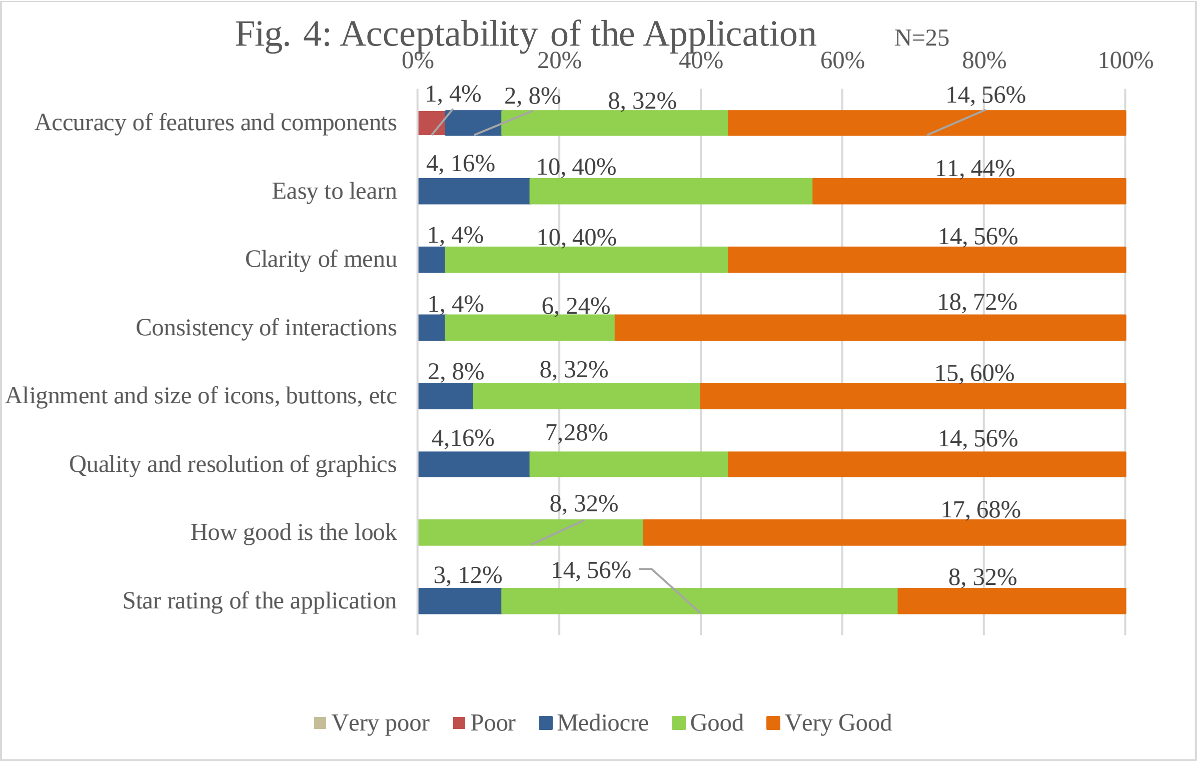
Fig. 4: Acceptability of the Application
N=25
0%
20%
40%
60%
80%
100%
Accuracy of features and components
1, 4%
2, 8%
8, 32%
14, 56%
Easy to learn
4, 16%
10, 40%
11, 44%
Clarity of menu
1, 4%
10, 40%
14, 56%
Consistency of interactions
1, 4%
6, 24%
18, 72%
Alignment and size of icons, buttons, etc
2, 8%
8, 32%
15, 60%
Quality and resolution of graphics
4,16%
7,28%
14, 56%
How good is the look
8, 32%
17, 68%
Star rating of the application
3, 12%
14, 56%
8, 32%
Very poor
Poor
Mediocre
Good
Very Good

Fig 5: Concerns regarding privacy and application related preferences

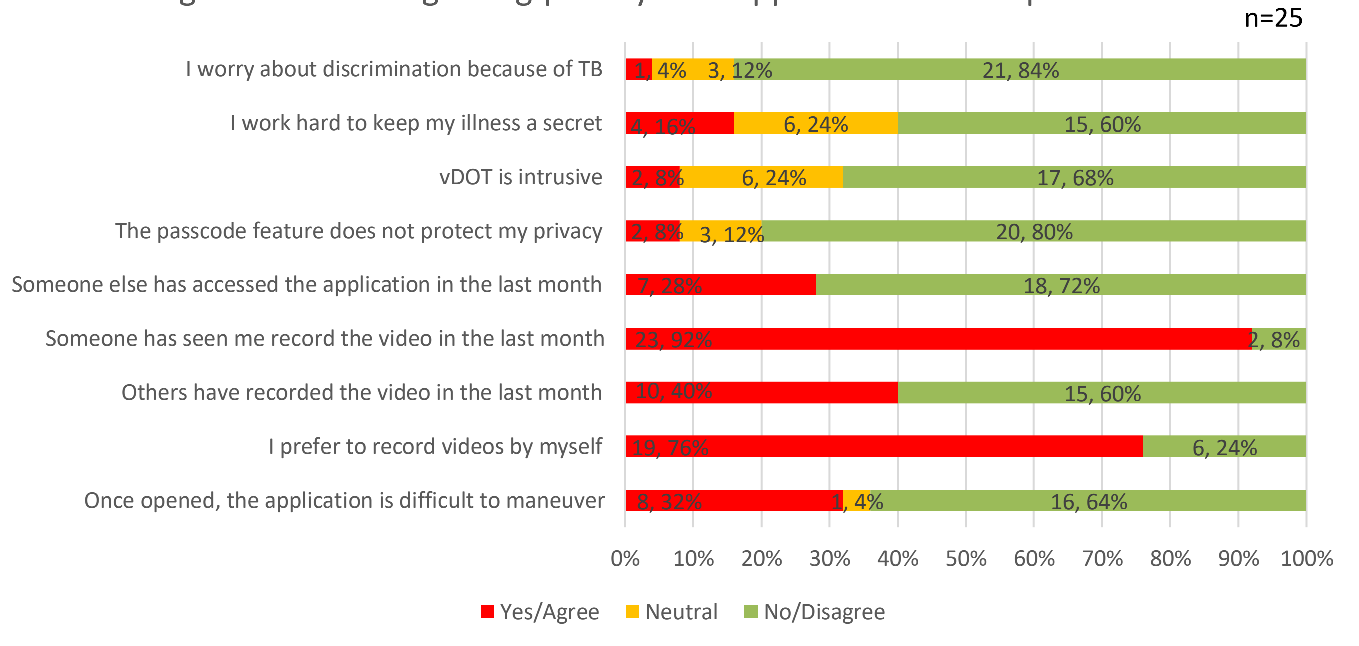

Table 1: Mixed-effects multilevel logistic regression analysis of the determinants of daily responsiveness during follow–up

Variable		Null Model	Logistic regression	Final Model
Fixed Effect Model		**Adjusted Odds Ratio (95% Confidence Interval)**	**Adjusted Odds Ratio (95% Confidence Interval)**	**Adjusted Odds Ratio (95% Confidence Interval)**
Sex	Females		0.99 (0.65-1.5)	0.55 (0.04 -8.36)
	Males		*Reference*	*Reference*
Age			1.04 (1.03 – 1.06)	1.04 (0.95 -1.14)
Area of residence	Urban		0.76 (0.09-6.43)	
	Rural		*Reference*	
Marital status	Married		5.09 (1.32 -19.65)	
	Unmarried		*Reference*	
Employment status	Employed		0.13 (0.06 -0.25)	0.01 (0.0003 – 0.31)
	Unemployed		*Reference*	*Reference*
Family income	Above Poverty Line		0.82 (0.54 – 1.26)	
	Below Poverty Line		*Reference*	
Literacy	Literate		0.18 (0.06 - 0.51)	0.04 (0.0007 -2.70)
	Illiterate		*Reference*	*Reference*
Owns a phone	Yes		2.16 (0.7 – 6.7)	
	No		*Reference*	
Site of TB	Pulmonary TB		0.42 (0.28 -0.63)	0.37 (0.03 -4.63)
	Extra Pulmonary TB		*Reference*	*Reference*
Patient Preference of vDOT	Prefers vDOT over DOT		40.08 (11.75 – 136.67)	
	Prefers DOT over vDOT		*Reference*	
Time from treatment initiation to enrolment			0.97 (0.97 – 1.06)	0.97 (0.95 – 1.14)
Random Effect Model				
At individual level				2.19 (1.48 - 3.26)
Intra class cluster coefficient (95%CI) at Individual level		0.70 (0.51 - 0.84)		0.59 (0.40 – 0.76)
Akaike Information Criterion (AIC)		1129	1446	1130

For peer review only

CONSORT 2010 checklist of information to include when reporting a randomised trial*

Section/Topic	Item No	Checklist item	Reported on page No
**Title and abstract**			
	1a	Identification as a randomised trial in the title	Not Applicable
	1b	Structured summary of trial design, methods, results, and conclusions (for specific guidance see CONSORT for abstracts)	Page 2
**Introduction**			
Background and objectives	2a	Scientific background and explanation of rationale	Page 3
	2b	Specific objectives or hypotheses	Page 3-4
**Methods**			
Trial design	3a	Description of trial design (such as parallel, factorial) including allocation ratio	Not applicable
	3b	Important changes to methods after trial commencement (such as eligibility criteria), with reasons	Not applicable
Participants	4a	Eligibility criteria for participants	Page 4
	4b	Settings and locations where the data were collected	Page 4
Interventions	5	The interventions for each group with sufficient details to allow replication, including how and when they were actually administered	Page 4
Outcomes	6a	Completely defined pre-specified primary and secondary outcome measures, including how and when they were assessed	Page 5
	6b	Any changes to trial outcomes after the trial commenced, with reasons	Not applicable
Sample size	7a	How sample size was determined	Not applicable
	7b	When applicable, explanation of any interim analyses and stopping guidelines	Not applicable
Randomisation:			
Sequence generation	8a	Method used to generate the random allocation sequence	Not applicable
	8b	Type of randomisation; details of any restriction (such as blocking and block size)	Not applicable
Allocation concealment mechanism	9	Mechanism used to implement the random allocation sequence (such as sequentially numbered containers), describing any steps taken to conceal the sequence until interventions were assigned	Not applicable
Implementation	10	Who generated the random allocation sequence, who enrolled participants, and who assigned participants to interventions	Not applicable

For peer review only

Blinding	11a	If done, who was blinded after assignment to interventions (for example, participants, care providers, those assessing outcomes) and how	Not applicable
	11b	If relevant, description of the similarity of interventions	Not applicable
Statistical methods	12a	Statistical methods used to compare groups for primary and secondary outcomes	Page 6
	12b	Methods for additional analyses, such as subgroup analyses and adjusted analyses	Not applicable
**Results**			
Participant flow (a diagram is strongly recommended)	13a	For each group, the numbers of participants who were randomly assigned, received intended treatment, and were analysed for the primary outcome	Page 6
	13b	For each group, losses and exclusions after randomisation, together with reasons	Page 6
Recruitment	14a	Dates defining the periods of recruitment and follow-up	Page 4
	14b	Why the trial ended or was stopped	Not applicable
Baseline data	15	A table showing baseline demographic and clinical characteristics for each group	Page 7
Numbers analysed	16	For each group, number of participants (denominator) included in each analysis and whether the analysis was by original assigned groups	Not applicable
Outcomes and estimation	17a	For each primary and secondary outcome, results for each group, and the estimated effect size and its precision (such as 95% confidence interval)	Page 7&8
	17b	For binary outcomes, presentation of both absolute and relative effect sizes is recommended	Not applicable
Ancillary analyses	18	Results of any other analyses performed, including subgroup analyses and adjusted analyses, distinguishing pre-specified from exploratory	Page 8
Harms	19	All important harms or unintended effects in each group (for specific guidance see CONSORT for harms)	Not applicable
**Discussion**			
Limitations	20	Trial limitations, addressing sources of potential bias, imprecision, and, if relevant, multiplicity of analyses	Page 11
Generalisability	21	Generalisability (external validity, applicability) of the trial findings	Page 11
Interpretation	22	Interpretation consistent with results, balancing benefits and harms, and considering other relevant evidence	Page 10&11
**Other information**			
Registration	23	Registration number and name of trial registry	CTRI/2017/07/ 009052
Protocol	24	Where the full trial protocol can be accessed, if available	Not available
Funding	25	Sources of funding and other support (such as supply of drugs), role of funders	Page 11

For peer review only

*We strongly recommend reading this statement in conjunction with the CONSORT 2010 Explanation and Elaboration for important clarifications on all the items. If relevant, we also recommend reading CONSORT extensions for cluster randomised trials, non-inferiority and equivalence trials, non-pharmacological treatments, herbal interventions, and pragmatic trials. Additional extensions are forthcoming: for those and for up to date references relevant to this checklist, see www.consort-statement.org.

For peer review only

For peer review only - http://bmjopen.bmj.com/site/about/guidelines.xhtml
